# Observations of a splitting ocean cyclone resulting in subduction of surface waters

**DOI:** 10.1126/sciadv.adu3221

**Published:** 2025-07-23

**Authors:** Leo Middleton, Weiguang Wu, T. M. Shaun Johnston, Daniel R. Tarry, J. Thomas Farrar, Pierre-Marie Poulain, Tamay M. Özgökmen, Andrey Y. Shcherbina, Ananda Pascual, Craig L. McNeill, Malek Belgacem, Maristella Berta, Kathleen Abbott, Alexandra Z. Worden, Fabian Wittmers, Alex Kinsella, Luca R. Centurioni, Verena Hormann, Eugenio Cutolo, Joaquín Tintoré, Simón Ruiz, Benjamín Casas, Helena Cheslack, CALYPSO Collaboration, Eric A. D’Asaro, Amala Mahadevan

**Affiliations:** ^1^ Woods Hole Oceanographic Institute, Woods Hole, MA 02543, USA.; ^2^ Scripps Institution of Oceanography, University of California, San Diego, La Jolla, CA 92037, USA.; ^3^ Applied Physics Labratory, University of Washington, Seattle, WA 98105, USA.; ^4^ Istituto Nazionale di Oceanografia e Geofisica Sperimentale, Sgonico, Friuli-Venezia Giulia 34010, Italy.; ^5^ Rosenstiel School of Marine and Atmospheric Science, University of Miami, Miami, FL 33149, USA.; ^6^ Instituto Mediterráneo de Estudios Avanzados, University of the Balearic Islands, Palma 07190, Spain.; ^7^ Consiglio Nazionale delle Ricerche–Istituto di Scienze Marine, Venizia, Italy.; ^8^ Marine Biological Labratory, Woods Hole, MA 02543, USA.; ^9^ Balearic Islands Coastal Observing and Forecasting System, Palma 07122, Spain.

## Abstract

Earth’s oceans contain large numbers of cyclonic eddies, 10 to 25 kilometers in diameter and unresolved in climate simulations. However, we lack observations of these features due to their small size and fast evolution. Here, we present in situ observations of one such cyclonic eddy with intense chlorophyll at its center as it spontaneously splits into two smaller cyclonic eddies over a few days. This splitting rapidly transports surface waters to depth, with sustained vertical velocities of 60 meters per day, primarily from the center of the eddy where carbon concentrations are largest, facilitating efficient transfer of phytoplankton carbon to depth, below the well-mixed, sunlit surface layer. We reproduce the splitting process in an idealized ocean model and find that splitting is controlled by the initial elliptical eddy shape, size, and intensity. Our observations uncover a mechanism for subduction in the upper ocean and highlight the need for quantifying its global prevalence.

## Teaser

In situ observations of a cyclonic ocean eddy as it spontaneously splits into two smaller eddies, bringing surface water to depth.

## INTRODUCTION

Cyclonic ocean eddies, 10 to 25 km in diameter, are observable with the naked eye from space, manifesting as spirals in the sunglitter reflected from the ocean surface (*1*). Notably, oceanographer and astronaut Paul Scully-Power commented, after the space shuttle Challenger mission in 1984, that these cyclonic spiral eddies “are perhaps the most fundamental entity in ocean dynamics at this scale” (*2*). Subsequent studies of submesoscales (i.e., 1 to 30 km) in the ocean have confirmed that there is indeed a pronounced asymmetry between the prevalence of cyclonic and anticyclonic eddies (*3*, *4*). Cyclonic eddies are often associated with dense water anomalies, where nutrient-rich water is elevated from depth into the photic zone and exposed to solar radiation (*5*–*7*). In nutrient-limited regions, this phenomenon can trigger phytoplankton blooms, often detectable as peaks in surface chlorophyll fluorescence when observed from satellite (*8*).

The exchange of properties, such as heat and CO_2_, between the ocean and atmosphere is vital for Earth’s climate and biosphere. Much of this exchange is controlled by the rate at which surface waters, able to interact with the atmosphere, move beneath the surface well-mixed layer, replaced by water from below, rich in nutrients (*6*, *9*, *10*). This movement of surface water to depth is our working definition of subduction throughout this paper. The edges of mesoscale (30 to 100 km) anticyclonic eddies have been implicated as important sites of subduction: The sharp lateral density gradients along the eddy edges are thought to create the necessary conditions for mixed-layer instabilities, which transport the light water vertically and laterally over the dense water, restratifying the density gradient (*11*). A parameterization of this process suggests that 25% of the total organic carbon export flux from the North Atlantic spring bloom could be explained by eddy-driven restratification (*12*) comparable to the amount exported via the sinking of particulate organic carbon (POC) (*13*).

Vertical velocities are enhanced at submesoscales, suggesting that subduction could occur at these scales through a multitude of processes, beyond just mixed-layer instabilities. However, the growth and decay of phytoplankton, the production and size distribution of POC (*14*), and its spatial distribution are also key to determining the contribution of eddies to the subduction of POC, as it is the covariance between vertical velocity and small (nonsinking) POC that determines its vertical advective flux (*15*, *16*). Here, we describe a different mechanism for eddy-driven carbon export in which a submesoscale eddy subducts POC in the process of splitting into two smaller eddies, coupling together a region of high carbon concentration with large vertical velocities.

The splitting of eddies has been observed at the mesoscale [O(100 km)] from satellite altimetry (*17*–*20*) and sometimes found to be associated with topographical interaction (*21*, *22*). However, the tendency of large-scale eddies is to merge, forming larger structures, giving rise to an up-scale energy transfer, where energy is moved to larger scales (*23*, *24*). At mesoscales, vertical velocities are typically weak, and so eddy splitting has not been implicated as a major source of vertical fluxes. However, at the submesoscale, gradients in horizontal velocity become much more intense, and eddy rotation becomes of a similar magnitude to Earth’s rotation, and as a result, vertical velocities are intensified, allowing substantial fluxes of heat and small POC to occur (*25*). The integrated global impact of mesoscale versus submesoscale fluxes depends on their prevalence and their intensity. Recent studies have demonstrated that submesoscales are prevalent where lateral buoyancy gradients and deep-mixed layers are present, particularly in winter time, leading to their dominant role in vertical heat fluxes (*26*).

**Fig. 1.**
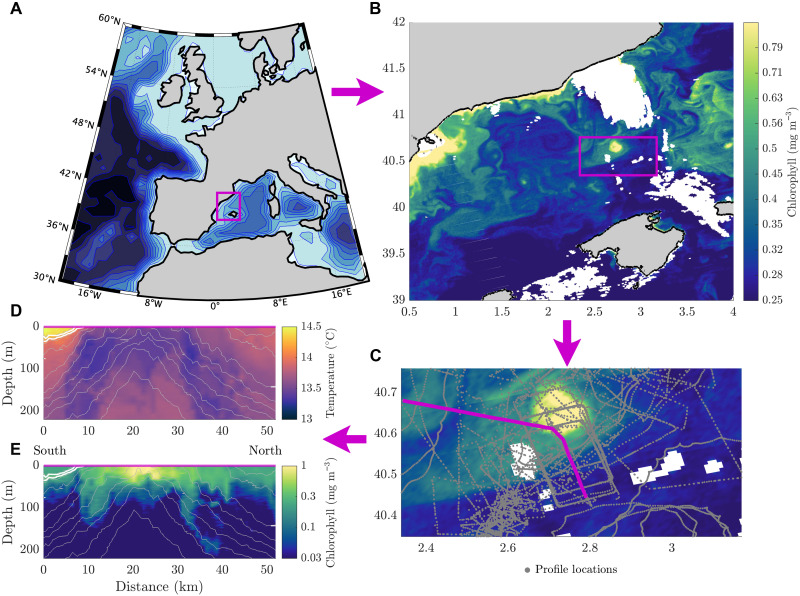
Location of measurements made during the 2022 CALYPSO Field Campaign. (**A**) Context map with magenta box showing the study region in the Mediterranean Sea. (**B**) Satellite chlorophyll a fluorescence image from NASA’s Visible Infrared Imaging Radiometer Suite (VIIRS) (*71*) on 23 February 2022. Magenta box inset shows the high-chlorophyll ocean cyclone considered in this study. (**C**) Same as (B) with profile locations during the study period marked in gray. Profiles from a sample section shown in (**D**) and (**E**) highlighted in magenta. Plotted against depth and alongtrack distance are the subsurface (D) temperature, (E) chlorophyll (in color), and potential density (contours) measured along a shiptrack [indicated in (C)] south to north through the eddy. The front in temperature to the south is outlined using white density contours in (D) and (E).

Here, we present in situ observations of a submesoscale ocean cyclone in the Mediterranean Sea that split into two distinct cyclones over a period of 5 days in February 2022. The observed eddy was identified on the basis of its anomalously high chlorophyll in satellite imagery (Fig. 1B). Subsurface observations revealed a rapid drop in density surfaces associated with the splitting, which suggests transport of the chlorophyll-rich surface water to depth. This has been verified with in situ water sampling. We subsequently developed an idealized model that captures the essence of this splitting behavior based only on the geometry of the initial observations of the eddy. The idealized model eddy splits on the same timescale as the observed eddy, and we obtain consistent estimates of the associated POC flux between observations and model, confirming that the splitting of submesoscale cyclones can provide a substantial flux of carbon below the photic zone. The idealized model captures prescient features of the observed splitting, so it allows us to analyze the mechanism underlying eddy splitting. We show that cyclogeostrophic balance, i.e., the force balance between the pressure gradient, Coriolis, and centrifugal forces, driven by the intense curvature of the flow around the narrow ends of the elliptically shaped eddy, can lead to a flow convergence that results in a positive feedback that intensifies the cyclonic vorticity in two distinct patches. These two regions of intensified cyclonic vorticity grow into the two 5-km eddies that result from the split. We explore a range of eddy intensities, sizes, and ellipticities within our idealized model to determine the conditions under which submesoscale eddies will split. The conditions required for splitting of elliptical eddies are commonly found at the submesoscale.

In the first section, we will describe the observations of the ocean cyclone and its subsequent splitting. In the second section, we will give details of the idealized model. Then, we will attempt to answer the questions: How do eddies split? When do eddies split? How much carbon transport does eddy splitting cause? Finally, we will discuss the potential implications of cyclone splitting for subduction in the upper ocean.

## RESULTS

### Observations

**Fig. 2.**
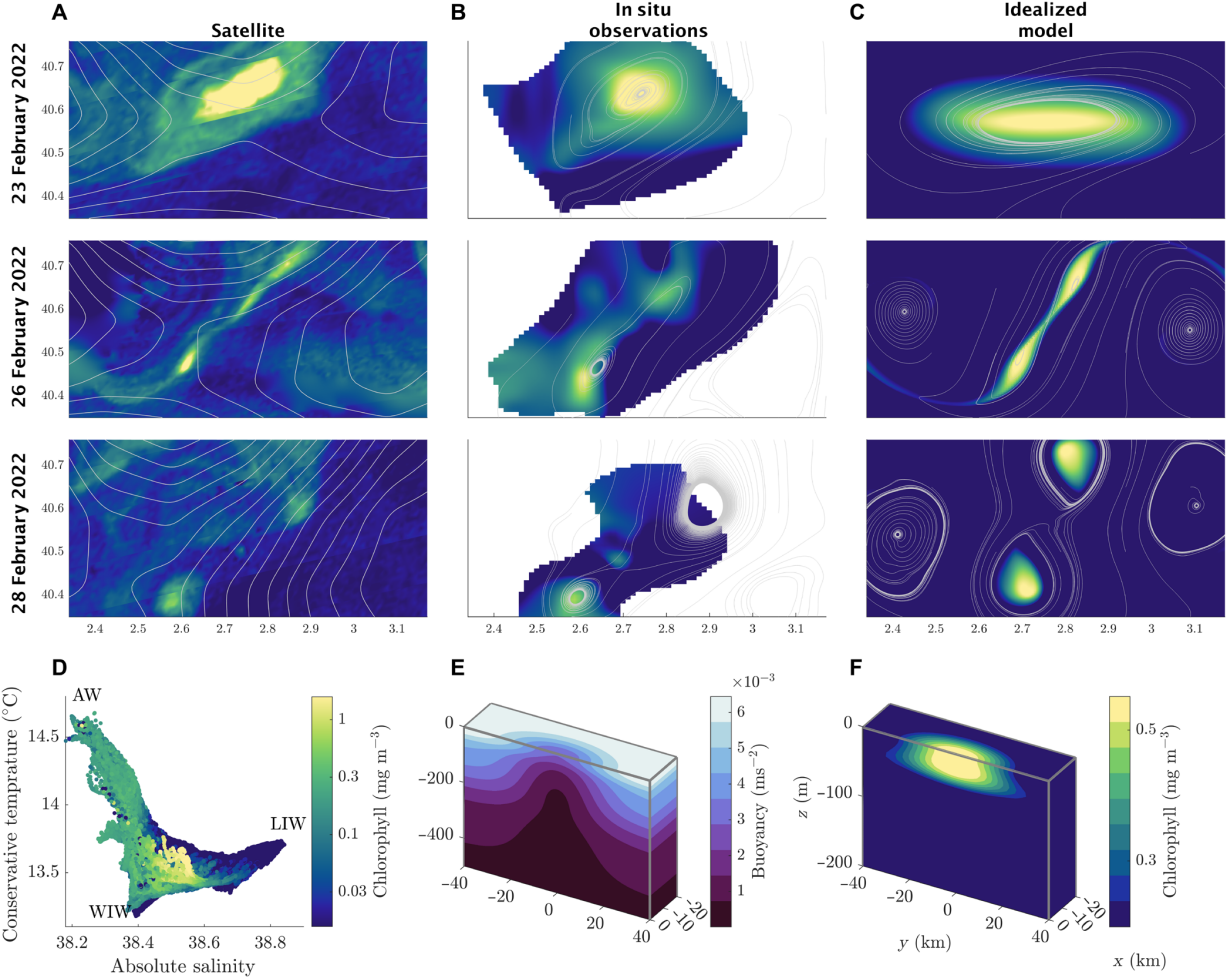
Surface evolution of high-chlorophyll ocean cyclone from multiple data sources, context on water masses, and model setup. (**A**) Time evolution of surface chlorophyll in images from NASA’s satellite VIIRS (*71*) and sea surface height anomaly contours in gray from NASA’s satellite Integrated Multi-Mission Ocean Altimeter Data for Climate Research (*72*), at three time points, showing the eddy thinning and splitting into two smaller eddies. (**B**) Chlorophyll a fluorescence at 20 m from a variational map of the observations (see Materials and Methods), at the same time points as the satellite data, with gray contours representing streamlines of the mapped velocity field. (**C**) Evolution of chlorophyll modeled as a passive tracer at the surface of an idealized model, with gray streamlines based on model velocities superimposed. The idealized model is initialized to match the observations using a Gaussian density distribution shown in (**E**). The passive tracer is initialized to match chlorophyll a concentrations in the observations, with a Gaussian shape shown in (**F**). (**D**) Temperature/salinity diagram of our observed vertical profiles, colored by chlorophyll. Three water masses are labeled: AW, LIW, and WIW.

Our observational program took place in the Balearic Sea, between mainland Spain and the Balearic Islands in the Mediterranean Sea (Fig. 1A). Although our field campaign took place in February, the observed mixed layers were very shallow, on average, less than 20 m (see the Supplementary Materials). During our observational campaign, we encountered a front between strongly stratified, low chlorophyll Atlantic Water (AW) from the south, and the weakly stratified, high-chlorophyll Winter Intermediate Water (WIW) (*27*). Beneath the surface, the density was primarily controlled by the high salinity Levantine Intermediate Water (LIW) (Fig. 2D) formed in the Eastern Mediterranean and transported to our region by eddies shed from the North Current, which carries modified waters from the Gulf of Lion to the Balearic Sea and has relatively high chlorophyll due to upwelling of nutrients along the northern boundary.

Between 24th February 2022 and the 1st March 2022, we intensively observed the evolution of a high-chlorophyll, 10-km radius, cyclonic eddy that was shed from the North current. During this period, the eddy elongated and stretched into a thin filament, visible in satellite chlorophyll (Fig. 2A), before splitting into two smaller cyclonic eddies. The more southern of the newly formed eddies (~5 km in radius) was tracked by our drifters and remained coherent for a period of weeks after the splitting event. The more northern of the two eddies was not as well sampled (Fig. 1C), which is why its chlorophyll signature does not appear as strongly in our mapped fields (Fig. 2B). These suggested dynamics from satellite are confirmed by our subsurface observations, illustrated by our mapped chlorophyll and velocity fields at 20-m depth shown in Fig. 2B. Our subsurface observations reveal a rapid drop in the density surfaces during the split, carrying surface water down with them. The dropping density surfaces suggest sustained vertical velocities of 60 m day^−1^, bringing oxygen- and carbon-rich surface water into the upper ocean, beneath the photic zone.

Our sampling plan was adaptive and responsive to our observations in real time, including data-assimilative regional ocean modeling and synoptic glider surveys. Sampling was achieved with underway profilers (*28*, *29*) that were repeatedly dropped (to ~250-m depth) from both ships to measure vertical profiles of temperature and salinity; one of these instruments, an EcoCTD (*29*), also measured chlorophyll fluorescence and oxygen subsurface during rapid surveys of the area (see Materials and Methods). The ships used a variety of acoustic doppler current profilers (ADCPs) to measure horizontal velocity as the ship moved, as detailed in Materials and Methods. From these ships, we deployed 9 profiling floats (*30*), 2 drifting and profiling Wirewalkers (*31*), and 106 drogued drifters (*32*–*35*).

Combining the subset of these data that fell within our study period, we produce a synoptic picture of the eddy-splitting process. To synthesize these large amounts of profiling data, we combine all 2812 profiles taken during the study period across the different instruments into a three-dimensional (3D) map of the splitting eddy, evolving with time. We use variational mapping (*36*, *37*) to create a smoothed map of density, eastward and northward velocities (*u* and *v*), chlorophyll fluorescence, and oxygen concentration. The mapped velocity is then used to calculate velocity gradients, including the vertical component of vorticity ζ.

**Fig. 3.**
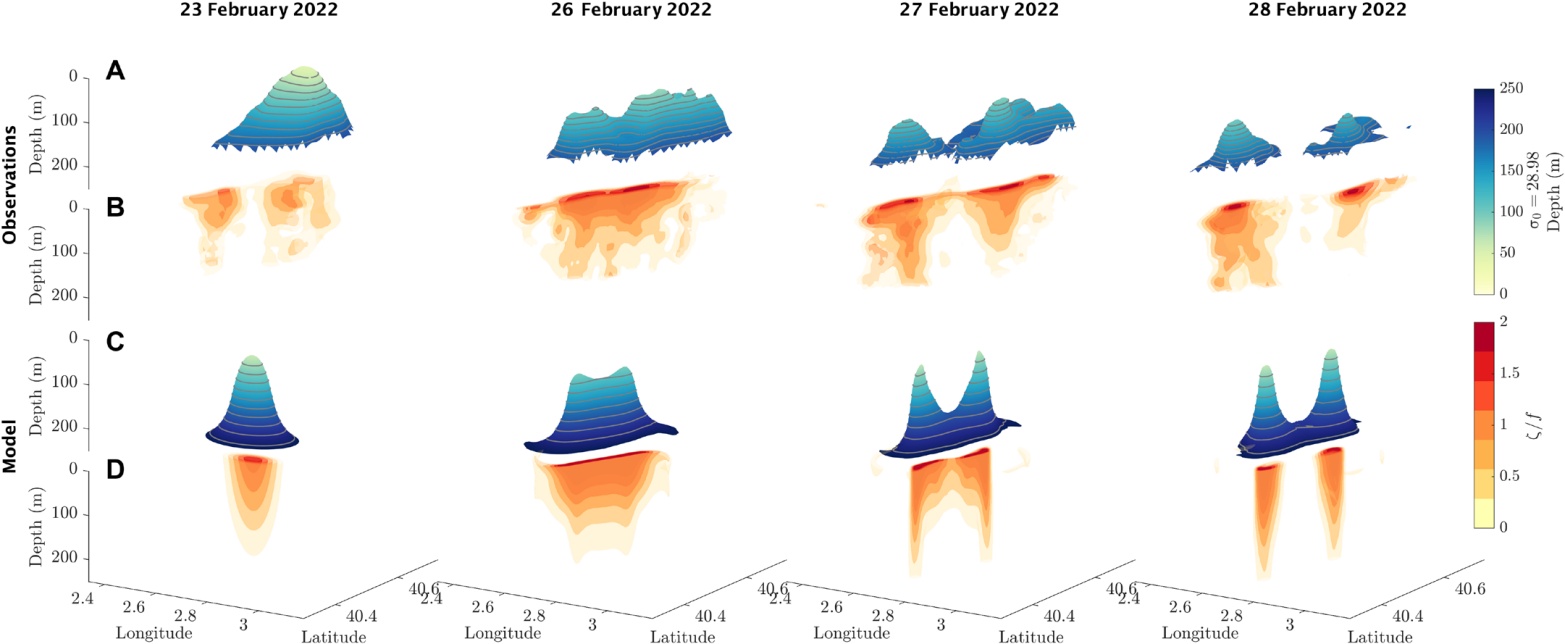
Subsurface evolution of the cyclonic eddy from an observational map and an idealized model, across four time points throughout the splitting process. (**A** and **C**) Evolution of surfaces of constant potential density σ_0_ = 28.98 kg m^−3^ (isopycnals) throughout the eddy splitting. Both color and elevation of the surface indicate the depth of the isopycnal. (**B** and **D**) Evolution of surfaces of constant vorticity $\zeta =\frac{\partial v}{\partial x}-\frac{\partial u}{\partial y}$ normalized by the Coriolis parameter *f*. Multiple vorticity surfaces are plotted, each colored by its normalized vorticity value. The first time point shows a single domed isoypcnal “hill”, with corresponding intensified vorticity at the center. The hill stretches into a “ridge” in the second time point, and vorticity intensifies and elongates. The center of the ridge drops in time points 3 and 4, and the vorticity separates into two separate cores, associated with two smaller domed isopycnals.

Over a period of 5 days, the cyclonic eddy, which had upward doming density surfaces (Fig. 3A) accompanied by a core of cyclonic vorticity subsurface (Fig. 3B) stretched and split. As the eddy elongated, the density surfaces were stretched into an elongated “ridge”-like structure (second column of Fig. 3). The center of the ridge then collapsed at an average rate of 60 m day^−1^ as the two smaller eddies intensified and separated. The vorticity intensified as the eddy elongated and extended deeper as the eddy split into two, leaving the two smaller cyclones with deeper cores of vorticity than the initial cyclone. These salient features of the observations (Fig. 3, A and B) are reproduced by our simplified idealized model (Fig. 3, C and D).

### Idealized model

There are multiple possible causes for the splitting of the observed eddy. Candidates include intense winds during the observed period that may have altered the surface vorticity balance; interactions between the eddy and the surface temperature front, visible in the section in Fig. 2D; or the influence of large-scale straining, as observed in the satellite sea surface height contours depicted in Fig. 2A. Instead, our findings indicate that the necessary conditions for the splitting process are just the initial shape of the eddy on 24 February, which is generically unstable. To show this, we conducted simulations of an idealized elliptical eddy in an ocean model, initialized to match the shape and size of the observed eddy (Fig. 2, E and F). Our modeled eddy reproduces the splitting process as shown in Fig. 2C, without the inclusion of winds, a front, or large-scale strain. The initial elliptical shape may be created by mesoscale strain, but once the eddy is elliptical, mesoscale strain is no longer required. In barotropic flows, it has been previously observed (*38*) that large-scale strain is not a necessary component to force cyclonic eddies to split. This observation is useful, as there are multiple potential causes for the elliptical shape of eddies within a fully developed field of eddies. The merging or interaction between distinct regions of vorticity (as observed in the initial stage of Fig. 3A) is another potential cause of ellipticity. The eddy formation mechanism itself may also cause eddies to become elliptical, as in “cat’s eye” pattern created by horizontal shear instability (*1*). Satellite observations show that elliptical shaped eddies are common at the submesoscale (*8*). Therefore, the conditions required for eddy splitting may be a natural part of eddy dynamics at these scales.

We initialized the idealized model using an elliptical Gaussian eddy, with stratification and initial vorticity consistent with our mapped observational fields. We assume the Coriolis parameter (*f*) is a constant due to the small spatial scale of our observations. We also initialized a passive tracer with an elliptical Gaussian distribution fit to the observed chlorophyll field. Cross sections of the model initial condition are shown in Fig. 2 (E and F). The idealized eddy elongates and splits on the same timescale as the observations and creates daughter eddies of a similar size and intensity to the observations (Fig. 3). In our idealized model, the elliptical eddy precesses about its axis, in keeping with a classic theory regarding elliptical eddies (*39*). However, in our observations, there is little evidence of eddy precession. We hypothesize that the lack of precession is due to the presence of a mesoscale strain field (apparent in sea surface height contours from satellite altimetry; Fig. 2A), which coincides with the observed temperature front. Although the exact details of the simulation and observations may differ, the model’s eddy splitting behavior, on time and length scales that are consistent with the observations, lead us to believe that we have successfully distilled the relevant aspects of the eddy-splitting process.

### How do eddies split?

In both the model and observations, the formation of two smaller eddies during the splitting process is due to an intensification of cyclonic vorticity in two distinct patches. This intensification can be thought of as a form of frontogenesis, where velocity gradients are increasing, along with buoyancy gradients, as seen in the thinning of the ridge in Fig. 3. It should be noted that nonsplitting elliptical eddies, discussed below, also undergo a similar frontogenetic intensification; however, in nonsplitting cases, the frontogenesis is arrested by the resymmetrizing of the eddy, rather than by splitting into two daughter eddies. Nevertheless, the frontogenetic process is an important precursor to splitting, so we have given a description of it here.

**Fig. 4.**
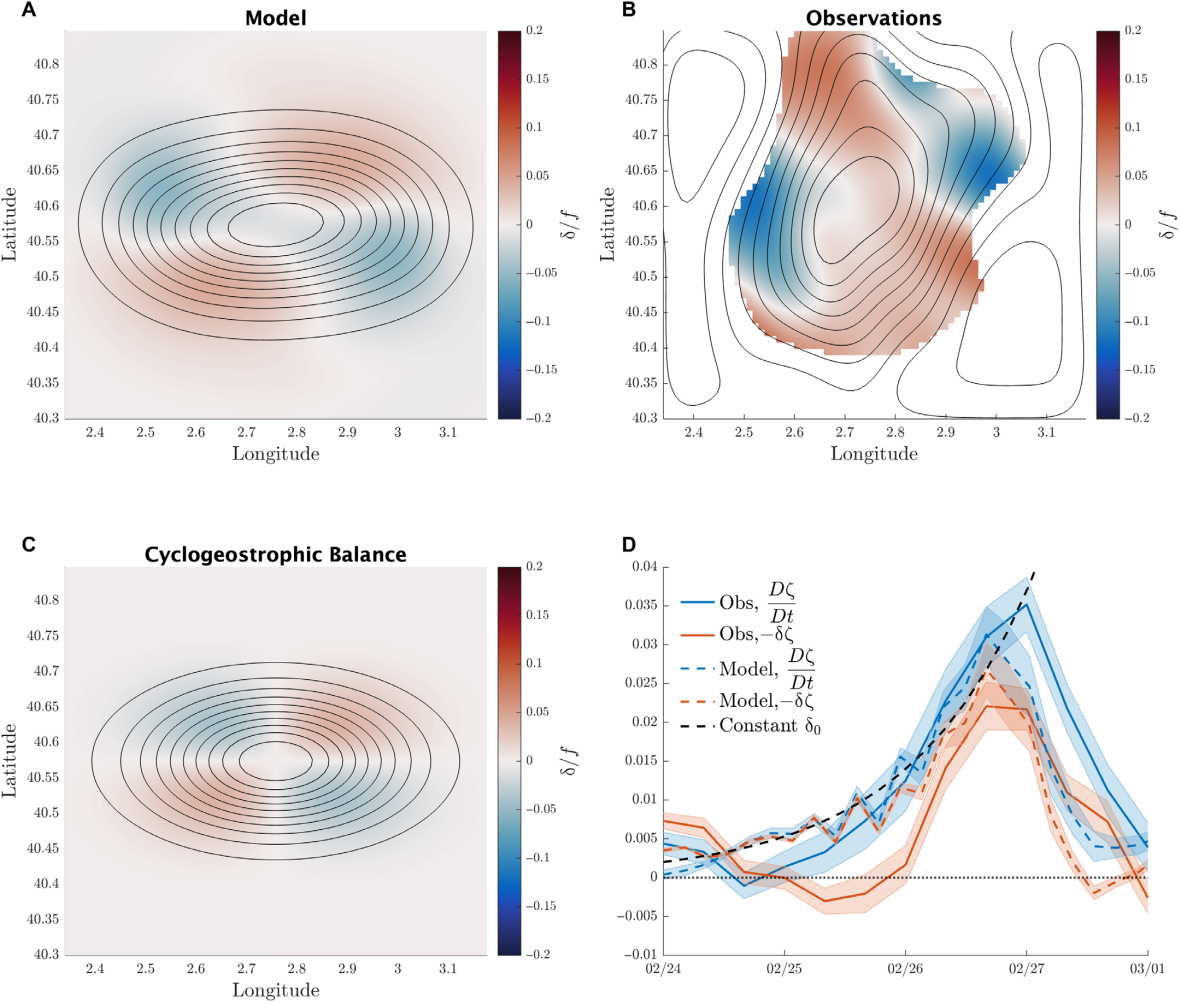
Demonstration of the typical velocity divergence patterns underlying the intensification that leads to eddy splitting. The pattern of divergence is similar between observations, model, and the predictions of cyclogeostrophic balance. Regions of convergence then lead to a feedback via vortex stretching that leads to an exponential growth in vorticity. (**A**) Normalized divergence δ/*f* smoothed spatially with a 10-km filter, averaged over the top 60 m of the idealized model and averaged over the first two inertial periods (37 hours) of the simulation. (**B**) Normalized divergence δ/*f* smoothed spatially with a 10-km filter, averaged over the upper 60 m of the variational map and over the first two inertial periods (37 hours) of the study period. (**C**) Normalized divergence δ/*f* calculated from cyclogeostrophic balance as applied to the idealized model initial condition, smoothed with a 10-km filter and averaged over the top 60 m. (**D**) Variation in time of the horizontally averaged rate of change of vorticity (blue) and the vortex stretching term (red) in both observations (thick lines) and idealized model (dashed lines) from Eq. 1. The theoretical prediction of a constant convergence is given with a black dashed line.

In our observational map and our idealized model, the evolution of vorticity is dominated by vortex stretching, so the approximate dynamical balance is given by

$$\underset{\begin{array}{c} \;\text{Rate}\;\text{of}\;\text{change}\;\\ \;\text{of}\;\text{vorticity}\; \end{array}}{\underbrace{\frac{D\zeta }{\mathrm{Dt}}}}\approx \underset{\begin{array}{c} \text{Vortex}\;\text{stretching} \end{array}}{\underbrace{-\delta \left(\zeta +f\right)}}$$ (1)

where the vorticity $\zeta =\frac{\partial v}{\partial x}-\frac{\partial u}{\partial y}$ and divergence $\delta =\frac{\partial u}{\partial x}+\frac{\partial v}{\partial y}$ . In Fig. 4D, we have plotted the domain averaged terms from the simulation evolving in time, which shows that changes in the vorticity are dominated by vortex stretching. Vortex stretching intensifies cyclonic (ζ > 0) vorticity in the presence of convergence. The presence of the Coriolis force creates an asymmetry, whereby cyclonic vorticity ζ > 0 is intensified, but anticyclonic vorticity ζ < 0 is not. The two smaller eddies that result from the split have intensifying cyclonic vorticity because they are associated with regions of convergence (ζ < 0). Vortex stretching is evident in the deepening of the core of vorticity of the smaller eddies (Fig. 3, B and D).

In Fig. 4, we have plotted the average pattern of normalized divergence δ/*f* in the model (Fig. 4A) and observations (Fig. 4B) averaged over the first day of evolution. The divergence forms a quadrupole pattern, with alternating patches of convergence and divergence around the eddy. The regions of convergence (δ < 0) are where the cyclonic vorticity preferentially intensifies due to vortex stretching, creating the two daughter eddies, resulting from the split (Fig. 3, B and D). Assuming a constant convergence δ_0_ = −0.1, we can solve Eq. 1 following a water parcel by integrating in time (*40*). This gives an exponential growth of vorticity in time

$$\zeta =\zeta_{0}e^{-\delta_{0}t}$$ (2)

following water parcels. We can show that this vorticity feedback does indeed occur by calculating the averaged rate of change of vorticity and vortex stretching, as defined above. If the vorticity is exponentially growing, so will these terms, without the need to follow water parcels. We have shown this growth in both the simulation and observations in Fig. 4D, as compared with the theoretical prediction of exponential growth.

The pattern of divergence in Fig. 4 (A and B) that causes vortex stretching and subsequent eddy splitting is due to the elliptical shape of the eddy. There is an additional centrifugal force that combines with the pressure gradient force to balance the Coriolis force in highly curved flows, creating what is known as cyclogeostrophic balance (*41*), an adjustment to the standard geostrophic balance. This can be written

$$\begin{array}{c} \underset{\begin{array}{c} \;\text{Centrifugal}\;\\ \;\text{force}\; \end{array}}{\underbrace{\rho_{0}\frac{V^{2}}{R}}}+\underset{\begin{array}{c} \;\text{Coriolis}\;\\ \;\text{force}\; \end{array}}{\underbrace{\rho_{0}\;\mathrm{fV}}}=\underset{\begin{array}{c} \;\text{Pressure}\;\text{gradient}\;\\ \;\text{force}\; \end{array}}{\underbrace{-\frac{\partial p}{\partial n}}} \end{array}$$ (3)

for the flow speed *V*, radius of curvature of streamlines *R*, the normal direction to the flow *n*, and pressure *p*. Note that this balance may hold with a varying *R* in complex flows and is not limited to circular eddies. For the model initial condition of an elliptical Gaussian eddy, we can derive a cyclogeostrophically balanced velocity from Eq. 3 and then calculate a divergence (Fig. 4C). The pattern and magnitude of the cyclogeostrophic divergence is similar to that of the full model (Fig. 4A), suggesting that cyclogeostrophic balance is responsible for the pattern of divergence. Unlike geostrophic balance, cyclogeostrophic balance is not necessarily divergence free. We can understand the quadrupole pattern in divergence (Fig. 4, A and B) by considering the acceleration of water as it circumnavigates the eddy. As the flow nears the narrow ends of the elliptical eddy, it slows down due to the additional compensation of the pressure gradient by the centrifugal force, creating a convergence. Then, as the flow passes the narrow end, it accelerates once more, creating a divergence.

Cyclogeostrophic balance does not capture the entire force balance, which can be seen in the differences between the initial model divergence and the cyclogeostrophically balanced divergence in Fig. 4 (A and C). However, the full velocity can be broken into a cyclogeostrophically balanced part and a noncyclogeostrophically balanced part, similar to the classic decomposition of velocity into geostrophic and ageostrophic components. The part of the velocity that is in cyclogeostrophic balance dominates enough of the divergence to explain the growth in vorticity via vortex stretching (Fig. 4D), even when using a constant value for the divergence δ_0_ from the initial cyclogeostrophic balance.

In the idealized model, internal gravity waves are initially radiated as the eddy adjusts from geostrophic balance to cyclogeostrophic balance (*42*). Then, as the eddy intensifies and splits, there is further radiation of internal gravity waves, as previously hypothesized in the case of a splitting mesoscale eddy (*20*). To average over unresolved internal waves in the divergence fields in Fig. 4 (A and B), we apply a spatial smoothing with a filter window of 10 km and average over twice the inertial period, given by 2π/*f* ≈ 18.5 hours, in both the observational map and the model.

The idealized model demonstrates that cyclogeostrophically balanced elliptical eddies have patterns of convergence and divergence due to the changes in speed implied by a strong centrifugal force at the two ends of the eddy. The convergence causes vortex stretching in two distinct patches. Feedback from the adjusting flow results in exponentially growing cyclonic vorticity and the creation of two smaller eddies. These two eddies then separate, leading to a drop in the isopycnal surface that originally connected them and completion of the splitting process.

### When do eddies split?

**Fig. 5.**
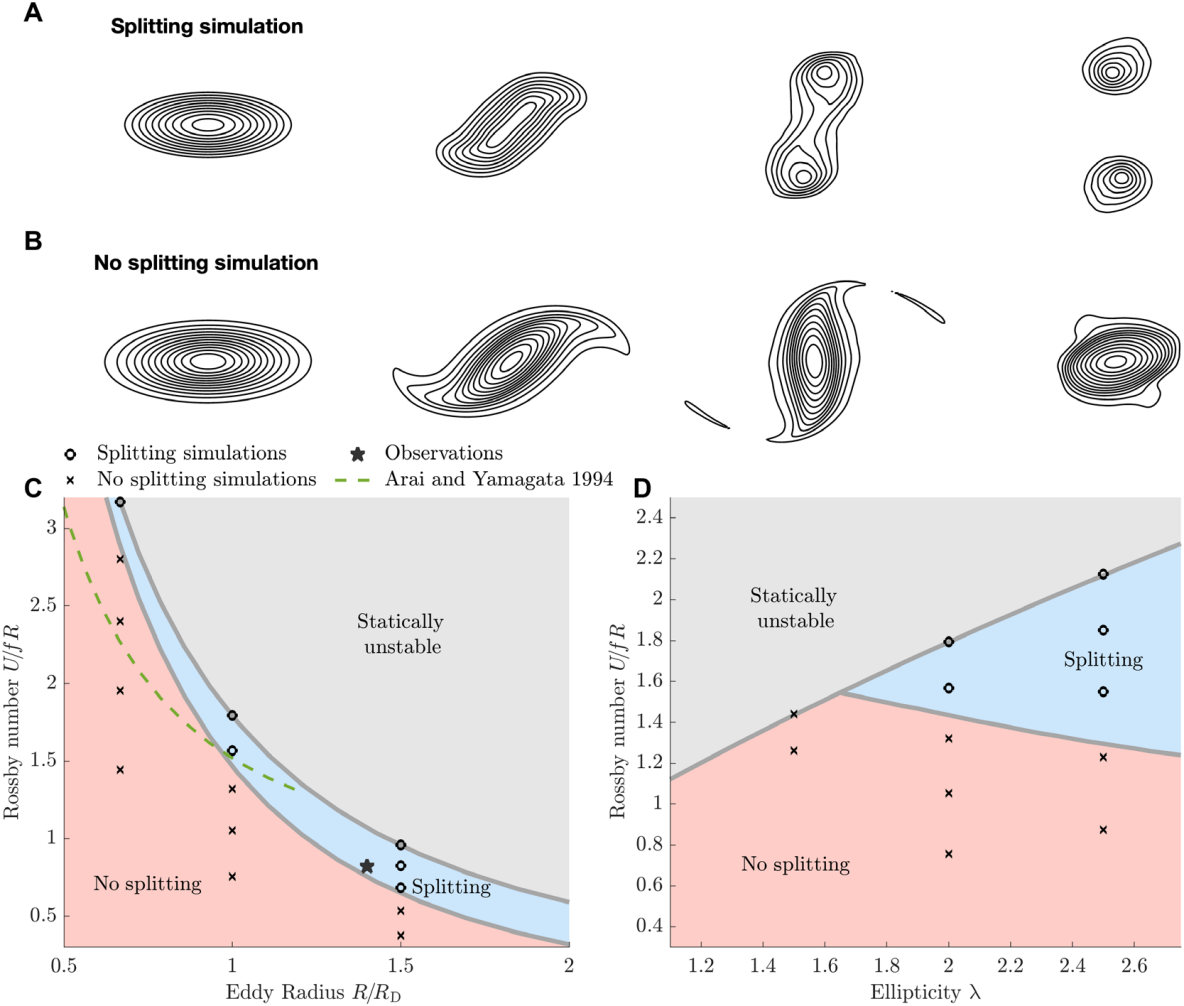
Schematic representation of the difference between splitting and nonsplitting eddies and mapping out the parameter space that distinguishes between the two cases. (**A** and **B**) Typical streamlines from idealized eddy simulations that show splitting and nonsplitting behaviors. (**C** and **D**) Parameter space in which the splitting/non-splitting simulations sit, marked with circles and crosses, respectively. (C) Variations in eddy radius *R* normalized by the radius of deformation *R*_D_, both defined in Materials and Methods, and the Rossby number *Ro* = *U*/*fR* for maximum eddy speed *U*, Coriolis parameter *f*, and eddy radius *R*. Regions where simulated eddies either split (blue) or do not split (red) are schematically colored. Parameter ranges where elliptical Gaussian eddies with exponentially decaying stratification are statically unstable (i.e., dense water on top of light water) are shaded in gray. The ellipticity of all simulations in (C) is λ = 2, and the parameters of the observed eddy are marked with a star. In (D), the eddy radius is held at a constant value of *R*/*R*_D_ = 1, so the observations do not sit in this cross section of parameter space.

We have observed how an initially elliptic eddy can split into two daughter eddies and shown with an idealized model that this behavior is a generic feature of the eddy shape, size, and intensity. However, not all elliptical eddies split. Cyclones commonly go through a process of resymmetrization, often discussed in the atmosphere to describe the behavior of tropical cyclones (*43*, *44*). By changing the initial conditions of the elliptical eddy in our idealized model, we can generate eddies that do not split. Example contours of sea surface height for simulated eddies that do not split are compared with those that do split in Fig. 5 (A and B). Nonsplitting eddies go through a similar process of elongation and intensification. However, instead of splitting into two daughter eddies, nonsplitting eddies collapse back into a single, more circular eddy. As the elliptical eddy rotates, it inhibits the intensification process, and vorticity filaments are shed from the edges, allowing the core to resymmetrize (*45*). Our observations do not show the same rotation as within the idealized model, so we may expect that the dynamics of resymmetrization make look somewhat different in the presence of large-scale strain.

The differences between splitting and resymmetrizing eddies have been previously explored for barotropic cyclones (*38*) using the rotating shallow water approximation. However, in our case, the dynamics are modified by the effects of a dynamically varying stratification using the full primitive equations (see Materials and Methods). In the shallow water equations (*38*), splitting was found empirically to occur for eddies with sufficiently large intensity or ellipticity. We find a qualitatively similar result, but to compare quantitatively to (*38*), we define the baroclinic Rossby radius of deformation *R*_D_ = *NH*/*f* for the buoyancy frequency $N=\sqrt{\frac{\partial b}{\partial z}}$ and the eddy height *H* (defined in Materials and Methods). Simulations with the same normalized eddy radius *R*/*R*_D_ and stratification profile will evolve in the same way, even if the actual radius *R* is modified. We define the eddy radius, *R*, as the geometric mean of the radii in the major and minor directions of the ellipse, assuming a Gaussian shape (see Materials and Methods). This does not give the radius of the outer-most eddy contour, so reported radius values are not the same as the maximum eddy radius. For example, the observed eddy fits a 10-km radius Gaussian eddy with an ellipticity of 2 despite the bowed isopycnals extending further than 10 km (Fig. 1, D and E). The radius of deformation in the observations is around 7 km, so we get a normalized radius of around 1.4.

We have run a series of simulations to determine which parameters are likely to result in splitting. We vary the eddy radius, normalized by the radius of deformation *R*_D_; the density anomaly associated with the eddy; and the ellipticity of the eddy. In Fig. 5C, we show simulations with a fixed ellipticity of λ = 2 but varying the eddy radius and density anomaly, and in Fig. 5D, we show simulations with a fixed eddy radius of *R*/*R*_D_ = 1 but varying the ellipticity. The eddy intensity can be measured using the Rossby number *Ro* = *U*/*fR* for maximum speed *U*, eddy radius *R*, and Coriolis parameter *f*. The stratification profile varies between the center of the eddy and the exterior. To ensure comparability across different parameter ranges, we used a simplified exponentially decaying stratification (see Materials and Methods) for simulations in which we vary the eddy parameters. One implication of this choice is that certain parameter ranges create a statically unstable density profile within the eddy (i.e., heavier water on top of lighter water), as the density changes horizontally at different rates across different depth levels. We have shaded out the statically unstable region within Fig. 5 (C and D).

For a given eddy radius, there is a critical threshold of intensity required to force splitting, as in the barotropic case (*38*). As you decrease the radius of the eddies, you need a higher critical intensity to tear them apart. At larger scales in the ocean, Rossby numbers rapidly decrease. So, we may not expect to find eddies of high-enough intensity at the mesoscale for splitting to occur. For example, Rossby numbers calculated from satellite altimetry in the Mediterranean Sea averaged at *Ro* = 0.05 for an average eddy radius of twice the radius of deformation (*46*) compared to the threshold for splitting of ~0.5 from our simulations for eddies that size. Submesoscales are defined by having order 1 Rossby numbers (*47*), and Rossby numbers substantially larger than 1 are often observed (*4*). Reaching the necessary Rossby number for a given eddy radius is therefore possible at the submesoscale, suggesting that eddy splitting may be more likely at these small scales.

Taking the analogous regime diagram from (*38*), we can compare directly their empirical prediction for the boundary of values that will force splitting to our own empirical fit. Figure 5C shows the comparison of the shallow water prediction, which fails to predict that our observed eddy would split, and overestimates the propensity to split for eddies with a small radius. From this comparison, we can infer that the missing physics from the shallow water equations could be important.

We also vary the ellipticity of the eddies while keeping the eddy radius constant (Fig. 5D). At small ellipticities, no splitting behavior was observed, across all statically stable Rossby numbers. Eddies that are sufficiently elliptical to split require high Rossby numbers to induce splitting. This suggests that in regions of large mesoscale strain, where elliptical-shaped eddies are more pronounced, we would expect more of them to undergo splitting. Ellipticity is a proxy for an eddy’s internal strain. Therefore, we might expect that eddies with a high internal strain rate to behave similarly to our elliptical Gaussian eddies as suggested by our observations.

It is important to note that the Fig. 5 (C and D) is regime diagram, rather than probability diagrams: The relatively small size of the splitting regime in this space is not indicative of a low probability of occurrence. Instead, to understand the prevalence of eddy splitting, we must also understand the distribution of Rossby numbers, eddy ellipticity, and deformation radii for submesoscale eddies in the global oceans. Quantifying these distributions and the prevalence of eddy splitting is beyond the scope of this manuscript. However, we have made steps toward understanding what would be required to assess the stability of submesoscale eddies to this form of instability.

In summary, using parameters representative of the ocean submesoscale in our model, we find that elliptical eddies split when they have a large Rossby number or, in other words, when they are particularly intense. The intensity required to split an eddy varies, depending on the size of the eddy measured relative to the eddy stratification and Earth’s rotation, quantified by the Rossby radius of deformation. Eddies also require a sufficient ellipticity to split; in our case, the ellipticity needs to exceed 1.5 for *R*/*R*_D_ = 1. We have marked the observed eddy in our regime space in Fig. 5C, where it suitably falls within the splitting region.

### How much carbon do splitting eddies transport?

**Fig. 6.**
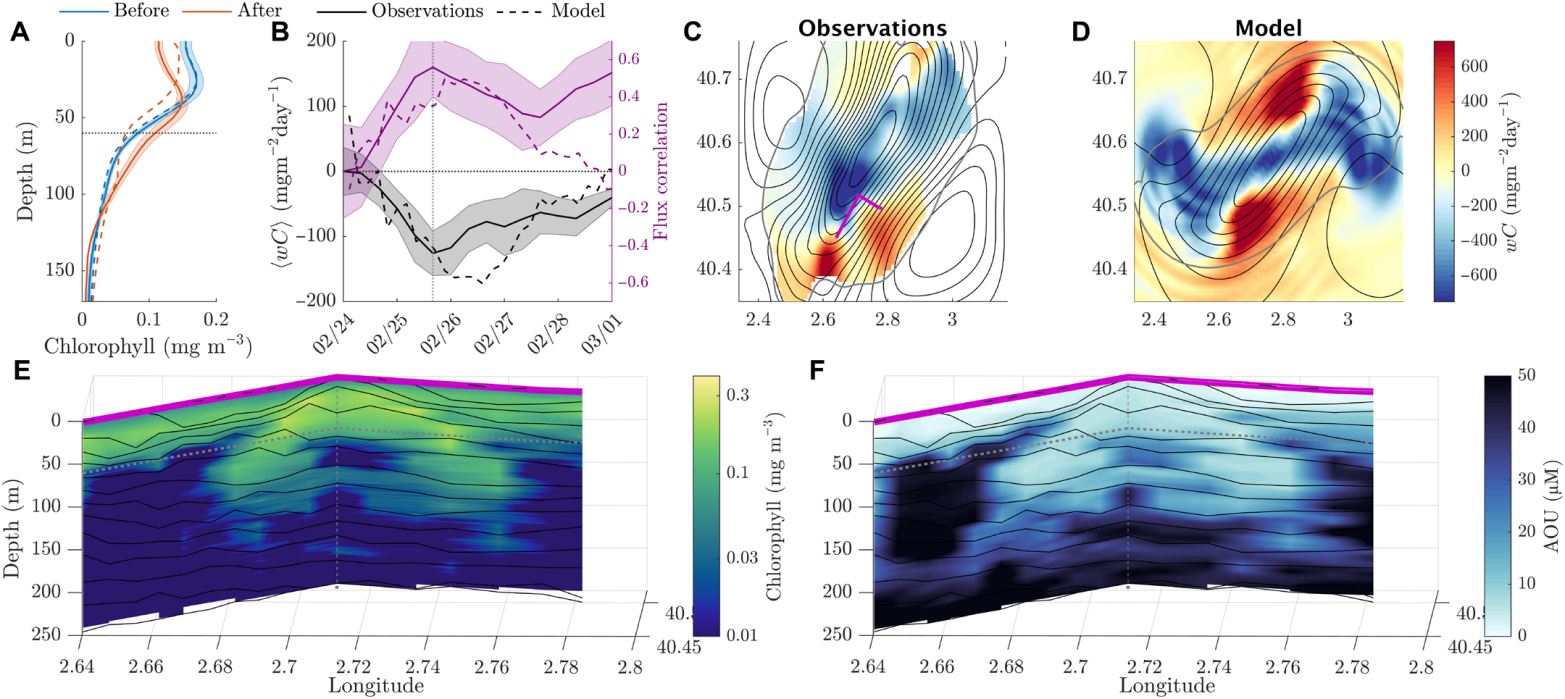
The contribution of eddy splitting to vertical fluxes of POC. (**A**) Horizontally averaged chlorophyll profile from the observations (thick lines) and the model (dashed lines), before (blue) and after (red) the eddy splitting. The horizontal dashed line marks the 60-m depth at which we present time and spatial flux variability. (**B**) Evolution of the POC flux (derived from chlorophyll, calibrated with water samples) from the observations (thick lines) and the model (dashed lines) at 60-m depth. We show the flux correlation $\langle \mathrm{wC}\rangle \;/\;\sqrt{\langle w^{2}\rangle \langle C^{2}\rangle }$ between the vertical velocity and chlorophyll in purple for model and observations. Error bars on the observational values in (A) and (B) are SEs derived from mapping uncertainty (**C**). (**D**) Snapshots of vertical POC flux from the observations and model respectively. Averaging regions are shown with a gray contour, and streamlines are shown with black contours. Magenta line shows location of example sections shown in (E) and (F). (**E**) Chlorophyll and (**F**) apparent oxygen utilization (AOU). Contours in (E) and (F) are of potential density.

As the eddy elongates, the domed isopycnals subsurface stretched into a ridge-like structure separating two regions of high vorticity (Fig. 3). During splitting, we observed that the density surfaces in the center of the ridge dropped, while the density surfaces were uplifted in the two daughter eddies as they formed. At the center of the ridge, where the chlorophyll concentrations were the largest, the isopycnal dropped the furthest, resulting in a transport of water from the surface to the interior. To show the potential effect of this transport on carbon fluxes, we plot the horizontally averaged chlorophyll profile before and after the splitting process in Fig. 6A. The average chlorophyll drops near the surface and increases at depth, in both the observations and model. This change is suggestive of vertical fluxes leading to a net subduction of chlorophyll and an obduction of lower-chlorophyll waters from below. However, horizontal advection, plankton photoacclimation, and biological production and respiration may play a role here. So, we estimate the local vertical POC flux 〈wC〉 implicated by the splitting, where *w* is the vertical velocity, *C* is the small POC concentration across the mapped region and within the model, and 〈⋅〉 is an average across the mapped region and within the corresponding buoyancy contour within the model, shown in Fig. 6.

To estimate the small POC concentration across the study region, we use chlorophyll fluorescence as a proxy. We took measurements of small POC from water samples during the cruise (see Materials and Methods) and then fit a curve *POC* = 64*chl*^0.3^ to the calibrated chlorophyll fluorescence (*48*). This proxy accounts for the effects of photoacclimation, which changes the carbon-to-chlorophyll ratio with depth and includes both living and dead (detrital) POC. We then use our map of chlorophyll a (Fig. 2) and an estimate of *w* to infer a flux of POC. To estimate the vertical velocity, we use the change in the height of the isopycnal. The isopycnal vertical velocity works well as a proxy for the full vertical velocity in the idealized model (see Materials and Methods). Alternative methods, such as the omega equation (*49*), require vertical velocity estimates in the near-surface layer as a boundary condition, which varies substantially at submesoscales (*50*). The assumptions of quasi-geostrophy, often required for the omega equation, are also strongly violated at the submesoscale. For example, the quadrupolar pattern of divergence in Fig. 4 would not be reproduced using the quasi-geostrophic approximation.

To understand the impact of eddy-subducted small POC, we report the flux at a given depth horizon. Water subducted beyond the mixed layer, beyond the photic zone, or beyond certain depth horizons will have qualitatively different effects on the biology and potential for re-entrainment into the surface layer. We choose to report vertical POC fluxes at 60 m as our depth horizon, below both the photic zone and the mixed layer. At 60 m, the photosynthetically available radiation (PAR) has fallen below 0.1% of the surface value, and there is no significant daily modulation of chlorophyll fluorescence (see the Supplementary Materials), so the effects of nonphotochemical quenching or biological production are limited.

In Fig. 6B, we show the horizontally averaged vertical flux of POC at 60 m depth as it varies in time, calculated from the observations and the idealized model. The vertical POC flux decreases as the eddy strains and intensifies before peaking as the eddy splits, reaching maximum values of 126 ± 24 mgC m^−2^ day^−1^ averaged across the map (Fig. 6B) and 992 ± 4 mgC m^−2^ day^−1^ locally (Fig. 6C). The spatial pattern of this flux shown in Fig. 6 (C and D) demonstrates the peak flux in the center of the splitting eddy, where the central isopycnal ridge drops, leading to large fluxes. In the model, there are also substantial fluxes along the edges of the eddy as it spins. However, we cannot be sure if these fluxes exist within our observations, as they are out of the range of our map, and the observed eddy does not spin substantially. For the observations, we only include segments of our map that have less than a 30% mapping error. To compare between the observations and model, we have averaged the vertical POC flux across a region of the simulations outlined in gray, defined using a buoyancy contour, chosen to cover similar buoyancy classes as those resolved in our observational map.

Further evidence of the substantial flux caused by the eddy splitting is provided by EcoCTD profiler data (*29*) taken after the splitting occurred. These transects profile through the region where the eddy’s center was previously located. We show transects of oxygen and calibrated chlorophyll fluorescence along with density contours. There was high chlorophyll a concentration and low apparent oxygen utilization observed at depth immediately after the splitting, well below the photic zone (see the Supplementary Materials). The low apparent oxygen utilization at depth is suggestive that physical subduction has occurred recently.

In summary, the splitting of a high-chlorophyll eddy in our observations is associated with large vertical fluxes of carbon, as estimated using interpolated chlorophyll fields, calibrated using measurements of POC, and using isopycnal height changes to estimate the vertical velocity. This carbon transport is quantitatively and qualitatively similar to the idealized model, where we do not have to rely on approximations for the vertical velocity. The average flux is also consistent with both the net change in the averaged chlorophyll profile and the observations of elevated chlorophyll and oxygen below the photic zone, directly after the splitting took place.

## DISCUSSION

Eddy splitting represents a coupled physical-biological mechanism for the subduction of carbon because there is a strong covariance between the region of elevated vertical velocity and the region of high chlorophyll (and POC) concentration. The center of the cyclonic eddy, with upward doming isopycnals, is a hotspot for phytoplankton productivity. The largest flux occurs during the splitting of the eddy due to the sinking of the ridge-like isopycnal surfaces formed by the stretching of the initial eddy. The center of the ridge is where the chlorophyll concentrations and vertical velocities were the largest. To illustrate this point, we have included a plot of the flux correlation $\langle \mathrm{wC}\rangle \;/\;\sqrt{\langle w^{2}\rangle \langle C^{2}\rangle }$ in Fig. 6B. The flux correlation shows that vertical velocities and chlorophyll concentration are highly correlated (0.56 ± 0.16 in the observations and 0.5 in the idealized model) during the splitting itself. After the eddy has split, there are large vertical velocities and fluxes around the resulting submesoscale coherent vortices (SCVs). However, the correlations between chlorophyll and vertical velocities are weaker after the split, so the motions are ineffective at delivering carbon to depth.

The horizontally averaged downward POC fluxes peak at 126 ± 24 mgC m^−2^ day^−1^ in the observations and 172 mgC m^−2^ day^−1^ in the model at 60-m depth, which is of a similar magnitude to scaling estimates of ~150 mgC m^−2^ day^−1^ during the North Atlantic spring bloom (*12*) at 100-m depth, attributed to mixed-layer eddy overturning. The maximum horizontally averaged small POC concentrations that we measured (~80 mgC) are similar to those in the North Atlantic (~40 to 100 mgC), suggesting that, depending on their rate of occurrence, splitting eddies could make a substantial contribution toward the averaged organic carbon flux comparable to that of mixed-layer instabilities. Although not discussed here, there are also a multitude of other mechanisms for subduction, likely present within our observations. The change in curvature of the flow path along the elliptical eddy also leads to subduction and is examined in an upcoming manuscript. Other mechanisms such as symmetric instability, nonlinear Ekman pumping, the interaction of near-inertial waves, and fronts of O(1) Rossby number are topics for future work.

We have not included any turbulent feedbacks, wind effects, or biological feedbacks within the idealized model, which may alter the flux estimates. Near-inertial internal waves are generated during splitting within the model, and if these result in locally increased turbulent mixing, then it that could enhance the downward flux of small POC at depth. There was a wind event during the eddy splitting (see the Supplementary Materials), which enhanced turbulent mixing in the surface mixed layer during the splitting. The mixed layer depth went from varying spatially between 0 and 20 m to between 20 and 50 m during the wind event, which enhanced the downward flux of small POC. The obduction of water from below the surface layer during splitting (Fig. 6, E and F), enriched in nutrients, may have led to enhanced biological production, increasing the pool of organic carbon available to be transported to depth. Recent work has also shown the importance of submesoscale dynamics to the export of medium-size POC (*14*), where the sinking speed is comparable to the vertical velocities at the submesoscale (~100 m day^−1^), suggesting that eddy splitting may also enhance the sinking POC flux. These possibilities allow for a substantially larger flux of carbon associated with eddy splitting than we have estimated.

Other tracers are also correlated with the occurrence of submesoscale cyclonic eddies, which may lead to enhanced fluxes during splitting. For instance, the eddy was associated with a cold temperature anomaly, and so splitting also induced a maximum heat flux of 34 ± 20 W m^−2^ (see the Supplementary Materials). This is comparable in magnitude to previous estimates from frontal regions (*49*), but the flux associated with the splitting of a given eddy will be heavily affected by the magnitude of heat anomalies associated with the eddy. Our observations were limited to 200 m; however in the idealized model, there was an increase in RMS vertical velocities during the split down to ~500 m in a 1000-m-depth model. We have not explored the potential impacts of eddy splitting at depth, but there is a deepening of the vorticity core of the daughter eddies during their formation due to vortex stretching (Fig. 3, B and D), which could lead to enhanced eddy stirring at depth. The evolution of the coherent eddies created by the splitting process are also not examined here but have implications for lateral transport of tracers.

We have only discussed the splitting process as it applies to cyclonic vortices. However, our observations point to a substantial asymmetry between cyclonic and anticyclonic splitting (*38*). For an anticyclonic split, the isopycnals would rise after the split, rather than drop, potentially leading to a net obduction, rather than subduction. This potential asymmetry in the effect of splitting eddies suggests a mechanism linking the cyclonic/anticyclonic asymmetry observed at submesoscales, particularly in the mixed layer (*3*), to the prevalence of subduction. Low potential vorticity anomalies (more anticyclonic) at depth are often thought to be indications of subduction; however, here, we have observed a high potential vorticity (more cyclonic) anomaly subducting. This is in keeping with theoretical and modeling studies where mixed layers are relatively shallow compared to the eddy scale (*51*, *52*).

At large scales in the ocean, there is an up-scale kinetic energy cascade, where eddies tend to merge, collecting motions into larger and larger structures, where kinetic energy is then dissipated primarily by bottom drag (*53*). At submesoscales on the other hand, we have the possibility of a downscale kinetic energy cascade, where energy is concentrated in successively smaller structures that are eventually dissipated by ocean turbulence and lastly viscosity. The downscale cascade has been directly observed using drifter measurements, and the transition occurs at ~10 km in active submesoscale flows (*54*). Naively, this downscale kinetic energy cascade could be interpreted as a tendency for eddies to split rather than to merge. However, previous studies on the downscale energy cascade have implicated sharp fronts as the primary regions of downscale energy transfer (*55*, *56*). Our observations suggest that eddy splitting could indeed play a role in the down-scale kinetic energy cascade: we have observed kinetic energy moving from the original eddy scale to the smaller scale of two daughter eddies. This is consistent with the downscale enstrophy cascade, i.e., vorticity distributions moving to smaller scales that happens at the mesoscale, but the coherence of the eddy structures that result from splitting implies kinetic energy has been transferred to the smaller structures. If eddy splitting is more likely at submesoscales, then it is possible that eddy splitting could play a role in the energy cascade, although further work is needed to quantify the contribution of eddy splitting to the average over a field of eddies.

Furthermore, we have shown that the conditions required to split an eddy are generic (moderate ellipticity, and high intensity relative to eddy radius), and potentially more likely at submesoscales, due to the high Rossby numbers that are possible in this range. However, in the splitting process we have outlined, the daughter-eddies themselves do not split, rather they remain coherent for an extended period of time. The lack of splitting of the daughter eddies is consistent with our characterization of splitting, as although they have high Rossby numbers, they do not have a substantial ellipticity, which is a necessary condition for splitting.

The two daughter eddies created by the splitting process are suitably identified with the term SCVs, which are small vorticies that remain coherent for long periods of time (months to years) (*57*). Many of the observed SCVs have been anticyclonic, and various formation mechanisms have been suggested (*58*, *59*). Here, we have demonstrated a formation mechanism for cyclonic SCVs, which we observed lasting for at least 2 months. These cyclonic SCVs may be responsible for substantial lateral transport of tracers, which is a topic for future study.

In describing the evolution of our idealized model and its path to splitting, we illustrated the importance of cyclogeostrophic balance. Previous authors have also implicated regions with heavily curved velocity fields as important sites of subduction (*60*–*62*). Highly curved velocity fields are a natural part of submesoscale dynamics due to their small spatial scales and large velocity gradients. Parameterizations of submesoscale subduction should account for the occurrence of highly curved regions, of which submesoscale eddy splitting is a prime example. While we do not quantify the frequency of occurrence of eddy splitting, we provide evidence that the conditions that permit this splitting are generic at the submesoscale. Future work is required to quantify the global frequency of eddy splitting and its large scale contribution to carbon fluxes.

## MATERIALS AND METHODS

### Instrumentation and processing

Two ships, the *N/O Pourquoi Pas?* and *R/V Pelagia* were used during the CALYPSO campaign for deployment of assets, water sampling, and underway profiling. Full accounts of the CALYPSO dataset, the instruments, and the cruise narrative are given in the cruise reports from the *N/O Pourquoi Pas?* (*63*) and *R/V Pelagia* (*64*). The *N/O Pourquoi Pas?* was equipped with 38-, 150-, and 300-kHz Teledyne ADCPs that measured down to 840-, 300-, and 120-m depths with resolutions of 12, 8, and 2 m, respectively. Underway profiling was achieved using the EcoCTD (810 profiles) and a traditional pumped CTD rosette system (24 profiles), which both measured conductivity, temperature, pressure, oxygen, and chlorophyll. The *R/V Pelagia* was equipped with a 75-kHz RDI ADCP measuring to 650-m depth with a resolution of 8 m. Underway profiling of conductivity and temperature was achieved with a mixture of Seabird and Valeport UCTD systems (870 profiles). Twenty-two SVP (*33*) drifters, 32 CODE (*34*) drifters, and 52 CARTHE (*35*) drifters were deployed and used as pointwise velocity measurements. We deployed two drifting and profiling WireWalker buoys (*31*), measuring conductivity and temperature and down to ~200-m depth, profiling every 20 min (553 profiles). One of the two WireWalkers also measured PAR, as reported in the Supplementary Materials. Also deployed were nine floats (eight SOLO-II and one Apex) measuring conductivity, temperature, and depth and profiling down to 250 m every hour (514 profiles).

For intercalibration, all instruments measuring conductivity, temperature, chlorophyll, or oxygen were referenced back to the CTD rosette system aboard the *N/O Pourquoi Pas?*, which was considered the gold standard due to its stable, high-quality sensors that were calibrated before and after the cruise to correct for any measurement drift. Simultaneous casts for calibration purposes were made where possible, and where not possible, we compared the most alike nearby casts in temperature/salinity space. Gain offsets were made to the conductivity cells where the differences were substantial (>0.01 mS cm^−1^) between the given instrument and the CTD rosette. Likewise, gain offsets were made to the EcoCTD oxygen and chlorophyll fluorescence sensors to align them with the CTD Rosette sensors.

### Water sampling

To make estimates of carbon fluxes, we directly measured POC using 49 water samples taken using Niskin bottles attached to the CTD Rosette. Four sets of triplicate samples were taken to constrain the measurement error across depths of 5 to 360 m. POC samples were collected on precombusted GF/F filters aboard the ship and then stored frozen at −20°C, before the filters were acid-fumed and dried at 60°C for 48 hours and then processed at the UCSB Analytical Laboratory using an automated organic elemental analyzer following the Dumas combustion method. All water samples used for calibration were taken during the night to avoid our calibration being biased by effects of nonphotochemical quenching. At all stages, samples were protected from light.

To calibrate the chlorophyll fluorescence optical measurements, we compared our near-surface samples to satellite estimates of chlorophyll concentration using a multisensor regional L3 ocean color product for the Mediterranean Sea. This gives chlorophyll values that are consistent with biogeochemical argo floats in the Balearic Sea and the repeat Mediterranean Ocean Observing System for the Environment (MOOSE) hydrographic glider section, which are both independently calibrated. We then derive a chlorophyll-to-POC ratio, as shown in the Supplementary Materials. The carbon flux estimates are independently calibrated with POC measurements, so are not sensitive to the calibration of the chlorophyll fluorescence sensor.

### Variational mapping

To create 3D spatial maps of the temperature, salinity, density, velocity, chlorophyll concentration, and oxygen concentration that evolve in time, we variational mapping using the program DIVAnd (Data-Interpolating Variational Analysis in n dimensions) (*36*). As an alternative approach to optimal interpolation [e.g. (*49*)], this method uses a minimization to find a 4D (3D + time) surface that is both smooth and close to the observations. Minimization allows for the application of physical constraints such as geostrophy (*37*), which we have not applied here, as we aim to capture ageostrophic splitting dynamics. We map the departure of the required fields from some constant background state, which we construct to mimic the front between the two water masses. We do this by taking the average profile for each variable in our initial surveys on 22 February, to the north and south of the front, and constructing a *tanh* function that smoothly moves between the two at each depth level. The mapping is insensitive to the orientation and slope of this chosen background state. To control the smoothness of the mapped surface and the proximity to observations, the user inputs two variables: a correlation scale in each dimension and a signal-to-noise ratio for the observations. Across the variety of sensors, the noise-to-signal ratio averages out at ~1%. The mapping technique allows for the estimation of mapping error covariances (*65*), which we propagate through the vertical velocity estimate and POC estimate to give a standard error for our mean flux measurements, shown with error bars in Fig. 6. The standard mapping error associated with our mean chlorophyll profiles are also given as error bars.

For the correlation lengths, some authors attempt an optimization over the data, withholding test data and optimizing for the best fit between the mapped data and the withheld data (*37*). However, in our case, we have specific dynamics (eddy splitting) that we are attempting to analyze, and so we choose our mapping based on the scales at which we would like to observe the dynamics. Specifically, one ship was maintaining a repeat section every 9 hours, half an inertial period, to allow for internal waves within our observations. Setting our time correlation scale to 9 hours then gives us natural choices for our spatial correlation lengths. We use a varying horizontal correlation length, chosen based on our estimate for velocity. For tracers being advected, we would expect the spatial correlation length to vary depending on the velocity. Using dimensional arguments, this suggests a spatial correlation length Δx=UΔt . This choice is similar to the Courant-Friedrichs-Lewy (CFL) condition in numerical methods (*66*) that provides a bound on the temporal resolution required to resolve waves of a certain wavelength. This condition gives a correlation length that varies in space and time throughout the map. We make a first guess of the correct correlation length by mapping the velocities with a constant horizontal correlation length of *L* = 5 km, and then using that estimate of velocities $\left[u_{0}\left(x,y,z,t\right),v_{0}\left(x,y,z,t\right)\right]$ , we set the correlation length in each direction for the final mapping as $\begin{array}{c} \left[L_{x},L_{y}\right]=\left[\;|\;u_{0}\left(x,y,z,t\right)\;|\Delta t,\;|\;v_{0}\left(x,y,z,t\right)\;|\Delta t\;\right] \end{array}$ for Δ*t* = 9 hours the temporal correlation scale. In the vertical, we have an average measurement resolution of 5 m, so we choose the vertical correlation length as 20 m to preserve our high vertical resolution, with some smoothing to prevent spurious overturns in density.

### Model setup

The idealized model solves the nonhydrostatic Boussinesq equations in a periodic box with a free surface on top and a no-flux boundary condition at the base. We use Oceananigans (*67*), which is based on the architecture of the Massachusetts Institute of Technology general circulation model (*68*), we used an adaptive timestep using the CFL condition and a fifth-order weighted essentially nonoscillatory (WENO) advection scheme (*69*). We use 48 vertical levels reaching down to 1000-m depth, staggered to provide greater surface resolution, from 35 to 1 m at the surface. Horizontally, we use 500-m resolution to fully resolve the resulting vortex motions. The initial condition is an idealized elliptical Gaussian eddy given below. In the vertical, we use a background vertical buoyancy profile fit to observations outside of the eddy and an eddy vertical buoyancy profile fit to observations within the eddy. We use the variational map detailed above to fit the idealized eddy parameters, giving an ellipticity of 2, an eddy radius of 10 km, and a buoyancy anomaly of 0.0035 m s^−2^. In the experiments where we vary the governing parameters (Fig. 5, C and D), we use an exponentially decaying buoyancy profile instead of the profile fit to observations.

The idealized Gaussian eddy with prescribed ellipticity can be described as a buoyancy anomaly, where velocity field *u* and *v* are constructed to be in thermal wind balance. The initial condition for buoyancy anomaly that we choose is

$$b\left(x,y,z,t\right)=b_{0}\left(z\right)+\Delta b\;b_{1}\left(z\right)\;\text{exp}\;\left(-\frac{x^{2}}{2\lambda_{1}^{2}R^{2}}-\frac{y^{2}}{2\lambda_{2}^{2}R^{2}}\right)$$ (4)

where

$$\begin{array}{c} b_{0}\left(z\right)=N^{2}H\;\text{exp}\;\left(\frac{z}{H}\right),b_{1}\left(z\right)=\text{exp}\;\left(\frac{z}{H}\right) \end{array}$$ (5)

for the idealized cases included in the regime diagram in Fig. 5 (C and D). Here, the buoyancy anomaly of the eddy is Δ*b*, the vertical eddy decay rate is *H*, the eddy radius is *R*, and the ellipticity is defined by λ, i.e., the radius in one direction divided by the radius in the other direction. Then, $\lambda_{1}=\sqrt{\lambda }$ and $\lambda_{2}=1/\sqrt{\lambda }$ are derived from the ellipticity λ. For the case matched to observations, $b_{0}\left(z\right)$ is the mean buoyancy profile across the mapped buoyancy field, and $b_{1}\left(z\right)$ is the mean buoyancy profile across the center of the eddy. To derive the parameters Δ*b*, *H*, *R*, and λ, we find the minimum root mean squared difference between the observational map of the eddy buoyancy and the idealized Gaussian model eddy above. We initialized the horizontal velocity using geostrophic balance; however, we experimented with initializations using cyclo-geostrophic balance and found qualitatively similar results. Our geostrophic initialization matched the observations more closely in terms of time taken to split, so we used this initialization for our intercomparison.

### Vertical velocities

To calculate vertical velocities that can be used for flux calculations, as in Fig. 6, we use the changes in isopycnal height in time (*49*). This relationship can be derived by starting with the equation for the evolution of buoyancy and transforming into thickness-weighted coordinates (*70*). Neglecting advection and diabatic effects, we can estimate

$$w=\frac{\partial h}{\partial t}+u_{h}\cdot \nabla h+\text{diffusion}\approx \frac{\partial h}{\partial t}$$ (6)

**Fig. 7.**
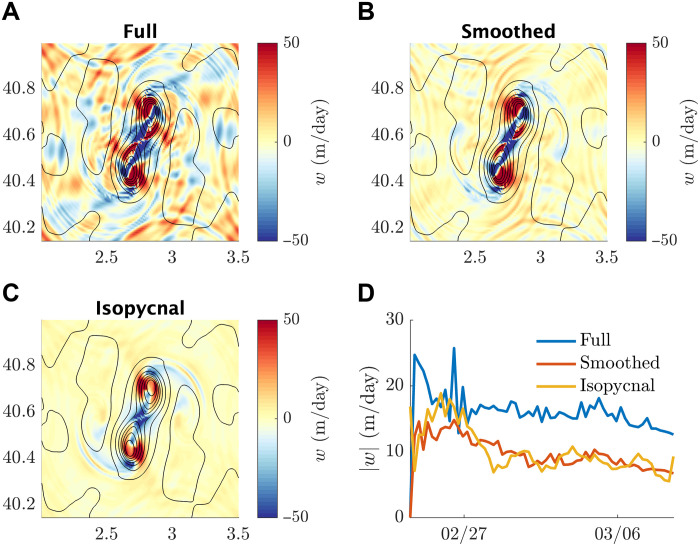
The efficacy of the isopycnal approximation of *w* within the idealized model. (**A**) Snapshot of the vertical velocity at 60-m depth on 28 February 2022. (**B**) Same as in (A) but with a 5-hour smoothing in time. (**C**) Snapshot of the isopycnal approximation to vertical velocity at 60-m depth on 28 February 2022. (**D**) Horizontal averaged vertical velocity magnitude ∣w∣ evolving through time, including the full velocity (blue), smoothed in time velocity (red), and isopycnal-only velocity (yellow).

To verify that our estimates of *w* are reasonable, we have plotted the true vertical velocity from the idealized model compared with the vertical velocity inferred from the isopycnal motion alone. In Fig. 7, we show cross sections of vertical velocity taken at 60 m and magnitude of the absolute vertical velocities averaged between 0 and 200 m to show the variability. After smoothing the velocity in time with a 5-hour window, we find a good agreement between the observed vertical velocity and the prediction based on isopycnal height. Considering the spatial variations, there are some minor differences, but the overall patterns are well captured.

## Supplementary Materials

### This PDF file includes:

CALYPSO Collaboration authors and affiliations Supplementary Materials and Methods Supplementary Text Figs. S1 to S3 References

## References

[1] W. Munk, L. Armi, K. Fischer, F. Zachariasen, Spirals on the sea. Proc. R. Soc. Lond. Ser. A: Math. Phys. Eng. Sci. 456, 1217–1280 (2000).

[2] P. Scully-Power, *Navy Oceanographer Shuttle Observations, STS 41-G Mission Report* (Naval Underwater Systems Center, NUSC Technical Document 7611, 1986).

[3] D. L. Rudnick, On the skewness of vorticity in the upper ocean. Geophys. Res. Lett. 28, 2045–2048 (2001).

[4] A. Y. Shcherbina, E. A. D’Asaro, C. M. Lee, J. M. Klymak, M. J. Molemaker, J. C. McWilliams, Statistics of vertical vorticity, divergence, and strain in a developed submesoscale turbulence field. Geophys. Res. Lett. 40, 4706–4711 (2013).

[5] H. Cao, M. Freilich, X. Song, Z. Jing, B. Fox-Kemper, B. Qiu, R. D. Hetland, F. Chai, S. Ruiz, D. Chen, Isopycnal submesoscale stirring crucially sustaining subsurface chlorophyll maximum in ocean cyclonic eddies. Geophys. Res. Lett. 51, e2023GL105793 (2024).

[6] P. Klein, G. Lapeyre, The oceanic vertical pump induced by mesoscale and submesoscale turbulence. Ann. Rev. Mar. Sci. 1, 351–375 (2009). 10.1146/annurev.marine.010908.163704 21141041

[7] D. J. McGillicuddy Jr., A. R. Robinson, D. A. Siegel, H. W. Jannasch, R. Johnson, T. D. Dickey, J. McNeil, A. F. Michaels, A. H. Knap, Influence of mesoscale eddies on new production in the Sargasso Sea. Nature 394, 263–266 (1998).

[8] Q. Ni, X. Zhai, C. Wilson, C. Chen, D. Chen, Submesoscale eddies in the south China Sea. Geophys. Res. Lett. 48, e2020GL091555 (2021).

[9] M. Lévy, P. J. S. Franks, K. S. Smith, The role of submesoscale currents in structuring marine ecosystems. Nat. Commun. 9, 4758 (2018). 30420651 10.1038/s41467-018-07059-3 PMC6232172

[10] A. Mahadevan, The impact of submesoscale physics on primary productivity of plankton. Ann. Rev. Mar. Sci. 8, 161–184 (2016). 10.1146/annurev-marine-010814-015912 26394203

[11] B. Fox-Kemper, R. Ferrari, R. Hallberg, Parameterization of mixed layer eddies. Part I: Theory and diagnosis. J. Phys. Oceanogr. 38, 1145–1165 (2008).

[12] M. M. Omand, E. A. D’Asaro, C. M. Lee, M. J. Perry, N. Briggs, I. Cetinić, A. Mahadevan, Eddy-driven subduction exports particulate organic carbon from the spring bloom. Science 348, 222–225 (2015). 25814062 10.1126/science.1260062

[13] P. W. Boyd, H. Claustre, M. Levy, D. A. Siegel, T. Weber, Multi-faceted particle pumps drive carbon sequestration in the ocean. Nature 568, 327–335 (2019). 30996317 10.1038/s41586-019-1098-2

[14] M. Dever, D. Nicholson, M. Omand, A. Mahadevan, Size-differentiated export flux in different dynamical regimes in the ocean. Global Biogeochem. Cycles 35, e2020GB006764 (2021).

[15] L. Resplandy, M. Lévy, D. J. McGillicuddy Jr., Effects of eddy-driven subduction on ocean biological carbon pump. Global Biogeochem. Cycles 33, 1071–1084 (2019).

[16] R. Asselot, L. I. Carracedo, V. Thierry, H. Mercier, R. Bajon, F. F. Pérez, Anthropogenic carbon pathways towards the North Atlantic interior revealed by Argo-O_2_, neural networks and back-calculations. Nat. Commun. 15, 1630 (2024). 38388482 10.1038/s41467-024-46074-5 PMC10884407

[17] W. Cui, W. Wang, J. Zhang, J. Yang, Multicore structures and the splitting and merging of eddies in global oceans from satellite altimeter data. Ocean Sci. 15, 413–430 (2019).

[18] Q.-Y. Li, L. Sun, S.-F. Lin, GEM: A dynamic tracking model for mesoscale eddies in the ocean. Ocean Sci. 12, 1249–1267 (2016).

[19] M. W. Schouten, W. P. de Ruijter, P. J. Van Leeuwen, J. R. Lutjeharms, Translation, decay and splitting of Agulhas rings in the southeastern Atlantic Ocean. J. Geophys. Res. Oceans 105, 21913–21925 (2000).

[20] D. Nof, The role of angular momentum in the splitting of isolated eddies. Tellus A Dyn. Meteorol. Oceanogr. 42, 469–481 (2022).

[21] H. L. Simmons, D. Nof, Islands as eddy splitters. J. Mar. Res. 58, 919–956 (2000).

[22] F. Fang, R. Morrow, Evolution, movement and decay of warm-core Leeuwin Current eddies. Deep Sea Res. II Top. Stud. Oceanogr. 50, 2245–2261 (2003).

[23] S. Wang, Z. Liu, C. Pang, Geographical distribution and anisotropy of the inverse kinetic energy cascade, and its role in the eddy equilibrium processes. J. Geophys. Res. Oceans 120, 4891–4906 (2015).

[24] J. M. Steinberg, S. T. Cole, K. Drushka, R. P. Abernathey, Seasonality of the mesoscale inverse cascade as inferred from global scale-dependent Eddy energy observations. J. Phys. Oceanogr. 52, 1677–1691 (2022).

[25] H. Hewitt, B. Fox-Kemper, B. Pearson, M. Roberts, D. Klocke, The small scales of the ocean may hold the key to surprises. Nat. Clim. Chang. 12, 496–499 (2022).

[26] Z. Su, J. Wang, P. Klein, A. F. Thompson, D. Menemenlis, Ocean submesoscales as a key component of the global heat budget. Nat. Commun. 9, 775 (2018). 29472586 10.1038/s41467-018-02983-w PMC5823912

[27] M. Juza, L. Renault, S. Ruiz, J. Tintoré, Origin and pathways of winter intermediate water in the northwestern mediterranean sea using observations and numerical simulation. J. Geophys. Res. Oceans 118, 6621–6633 (2013).

[28] D. L. Rudnick, J. Klinke, The underway conductivity–temperature–depth instrument. J. Atmos. Oceanic Tech. 24, 1910–1923 (2007).

[29] M. Dever, M. Freilich, J. T. Farrar, B. Hodges, T. Lanagan, A. J. Baron, A. Mahadevan, EcoCTD for profiling oceanic physical–biological properties from an underway ship. J. Atmos. Oceanic Tech. 37, 825–840 (2020).

[30] R. Davis, J. Sherman, J. Dufour, Profiling ALACEsdd other advances in autonomous subsurface floats. J. Atmos. Oceanic Tech. 18, 982–993 (2001).

[31] R. Pinkel, M. Goldin, J. Smith, O. Sun, A. Aja, M. Bui, T. Hughen, The Wirewalker: A vertically profiling instrument carrier powered by ocean waves. J. Atmos. Oceanic Tech. 28, 426–435 (2011).

[32] P.-M. Poulain, L. Centurioni, T. Özgökmen, Comparing the currents measured by CARTHE, CODE and SVP drifters as a function of wind and wave conditions in the southwestern Mediterranean Sea. Sensors 22, 353 (2022). 35009913 10.3390/s22010353 PMC8749831

[33] R. Lumpkin, M. Pazos, Measuring surface currents with Surface Velocity Program drifters: The instrument, its data, and some recent results. LAPCOD 39, 67 (2007).

[34] R. E. Davis, Drifter observations of coastal surface currents during CODE: The method and descriptive view. J. Geophys. Res. Oceans 90, 4741–4755 (1985).

[35] G. Novelli, C. M. Guigand, C. Cousin, E. H. Ryan, N. J. Laxague, H. Dai, B. K. Haus, T. M. Özgökmen, A biodegradable surface drifter for ocean sampling on a massive scale. J. Atmos. Oceanic Tech. 34, 2509–2532 (2017).

[36] A. Barth, J.-M. Beckers, C. Troupin, A. Alvera-Azcárate, L. Vandenbulcke, divand-1.0: *n*-dimensional variational data analysis for ocean observations. Geosci. Model Dev. 7, 225–241 (2014).

[37] E. Cutolo, A. Pascual, S. Ruiz, T. Shaun Johnston, M. Freilich, A. Mahadevan, A. Shcherbina, P.-M. Poulain, T. Ozgokmen, L. R. Centurioni, D. L. Rudnick, E. D’Asaro, Diagnosing frontal dynamics from observations using a variational approach. J. Geophys. Res. Oceans 127, e2021JC018336 (2022).

[38] M. Arai, T. Yamagata, Asymmetric evolution of eddies in rotating shallow water. Chaos 4, 163–175 (1994). 12780097 10.1063/1.166001

[39] S. Kida, Motion of an elliptic vortex in a uniform shear flow. J. Physical Soc. Japan 50, 3517–3520 (1981).

[40] J. Dingwall, T. Chor, J. R. Taylor, Large eddy simulations of the accumulation of buoyant material in oceanic wind-driven and convective turbulence. J. Fluid Mech. 954, A27 (2023).

[41] J. R. Holton, G. J. Hakim, *An introduction to dynamic meteorology* (Academic press, 2012).

[42] C. J. Shakespeare, Curved density fronts: Cyclogeostrophic adjustment and frontogenesis. J. Phys. Oceanogr. 46, 3193–3207 (2016).

[43] D. A. Schecter, M. T. Montgomery, On the symmetrization rate of an intense geophysical vortex. Dyn. Atmos. Oceans 37, 55–88 (2003).

[44] C. Gao, P. Zhu, Vortex Rossby wave propagation in baroclinic tropical cyclone-like vortices. Geophys. Res. Lett. 43, 12–578 (2016).

[45] M. Melander, J. McWilliams, N. Zabusky, Axisymmetrization and vorticity-gradient intensification of an isolated two-dimensional vortex through filamentation. J. Fluid Mech. 178, 137–159 (1987).

[46] A. Ioannou, A. Stegner, A. Tuel, B. LeVu, F. Dumas, S. Speich, Cyclostrophic corrections of AVISO/DUACS surface velocities and its application to mesoscale eddies in the Mediterranean Sea. J. Geophys. Res. Oceans 124, 8913–8932 (2019).

[47] J. R. Taylor, A. F. Thompson, Submesoscale dynamics in the upper ocean. Annu. Rev. Fluid Mech. 55, 103–127 (2023).

[48] H. Loisel, A. Morel, Light scattering and chlorophyll concentration in case 1 waters: A reexamination. Limnol. Oceanogr. 43, 847–858 (1998).

[49] D. L. Rudnick, N. D. Zarokanellos, J. Tintoré, A four-dimensional survey of the Almeria–Oran Front by underwater gliders: tracers and circulation. J. Phys. Oceanogr. 52, 225–242 (2022).

[50] D. R. Tarry, S. Ruiz, T. S. Johnston, P.-M. Poulain, T. Özgökmen, L. R. Centurioni, M. Berta, G. Esposito, J. T. Farrar, A. Mahadevan, Drifter observations reveal intense vertical velocity in a surface ocean front. Geophys. Res. Lett. 49, e2022GL098969 (2022).

[51] M. A. Spall, Frontogenesis, subduction, and cross-front exchange at upper ocean fronts. J. Geophys. Res. Oceans 100, 2543–2557 (1995).

[52] M. Freilich, A. Mahadevan, Coherent pathways for subduction from the surface mixed layer at ocean fronts. J. Geophys. Res. Oceans 126, e2020JC017042 (2021).

[53] R. Ferrari, C. Wunsch, Ocean circulation kinetic energy: Reservoirs, sources, and sinks. Annu. Rev. Fluid Mech. 41, 253–282 (2009).

[54] D. Balwada, J.-H. Xie, R. Marino, F. Feraco, Direct observational evidence of an oceanic dual kinetic energy cascade and its seasonality. Sci. Adv. 8, eabq2566 (2022). 36223461 10.1126/sciadv.abq2566 PMC9555769

[55] K. Srinivasan, R. Barkan, J. C. McWilliams, A forward energy flux at submesoscales driven by frontogenesis. J. Phys. Oceanogr. 53, 287–305 (2023).

[56] M. Freilich, L. Lenain, S. T. Gille, Characterizing the role of non-linear interactions in the transition to submesoscale dynamics at a dense filament. Geophys. Res. Lett. 50, e2023GL103745 (2023).

[57] J. C. McWilliams, Submesoscale, coherent vortices in the ocean. Rev. Geophys. 23, 165–182 (1985).

[58] D. McCoy, D. Bianchi, A. L. Stewart, Global observations of submesoscale coherent vortices in the ocean. Prog. Oceanogr. 189, 102452 (2020).

[59] E. A. D’Asaro, Generation of submesoscale vortices: A new mechanism. J. Geophys. Res. Oceans 93, 6685–6693 (1988).

[60] J. A. MacKinnon, H. L. Simmons, J. Hargrove, J. Thomson, T. Peacock, M. H. Alford, B. I. Barton, S. Boury, S. D. Brenner, N. Couto, S. L. Danielson, E. C. Fine, H. C. Graber, J. Guthrie, J. E. Hopkins, S. R. Jayne, C. Jeon, T. Klenz, C. M. Lee, Y. D. Lenn, A. J. Lucas, B. Lund, C. Mahaffey, L. Norman, L. Rainville, M. M. Smith, L. N. Thomas, S. Torres-Valdés, K. R. Wood, A warm jet in a cold ocean. Nat. Commun. 12, 2418 (2021). 33893280 10.1038/s41467-021-22505-5 PMC8065036

[61] C. E. Buckingham, J. Gula, X. Carton, The role of curvature in modifying frontal instabilities. Part II: Application of the criterion to curved density fronts at low Richardson numbers. J. Phys. Oceanogr. 51, 317–341 (2021).

[62] E. Pallàs-Sanz, T. Johnston, D. Rudnick, Frontal dynamics in a California current system shallow front: 2. Mesoscale vertical velocity. J. Geophys. Res. Oceans 115, C12068 (2010).

[63] A. Mahadevan, E. A. D’Asaro, Cruise report: Calypso, R/V Pourquoi Pas? (OSF, 2022).

[64] T. M. S. Johnston, N. Calafat, B. Casas, E. Cutolo, R. Daniels, F. Falcieri, T. Litchendorf, I. Lizarán, C. McNeil, A. Pascual, Cruise report: Calypso, R/V Pelagia, Cruise No. 64pe497 (OSF, 2022); doi:10.17605/OSF.IO/EN7T4.

[65] C. Troupin, A. Barth, D. Sirjacobs, M. Ouberdous, J.-M. Brankart, P. Brasseur, M. Rixen, A. Alvera-Azcárate, M. Belounis, A. Capet, Generation of analysis and consistent error fields using the Data Interpolating Variational Analysis (DIVA). Ocean Model. 52, 90–101 (2012).

[66] C. A. De Moura, C. S. Kubrusly, The courant–friedrichs–lewy (cfl) condition. AMC 10, (2013).

[67] A. Ramadhan, G. Wagner, C. Hill, J.-M. Campin, V. Churavy, T. Besard, A. Souza, A. Edelman, R. Ferrari, J. Marshall, Oceananigans. jl: Fast and friendly geophysical fluid dynamics on GPUs. J. Open Sourc. Softw. 5, (2020).

[68] J. Marotzke, R. Giering, K. Q. Zhang, D. Stammer, C. Hill, T. Lee, Construction of the adjoint MIT ocean general circulation model and application to Atlantic heat transport sensitivity. J. Geophys. Res. Oceans 104, 29529–29547 (1999).

[69] X.-D. Liu, S. Osher, T. Chan, Weighted essentially non-oscillatory schemes. J. Comput. Phys. 115, 200–212 (1994).

[70] W. R. Young, An exact thickness-weighted average formulation of the Boussinesq equations. J. Phys. Oceanogr. 42, 692–707 (2012).

[71] Visible and Infrared Imager/Radiometer Suite (VIIRS), NASA goddard space flight center, ocean ecology laboratory, ocean biology processing group. (NASA OB.DAAC, 2022).

[72] B. D. Beckley, N. P. Zelensky, S. A. Holmes, F. G. Lemoine, R. D. Ray, G. T. Mitchum, S. D. Desai, S. T. Brown, Assessment of the Jason-2 extension to the TOPEX/Poseidon, Jason-1 sea-surface height time series for global mean sea level monitoring. Mar. Geod. 33, 447–471 (2010).

[73] E. Marañón, F. Van Wambeke, J. Uitz, E. S. Boss, M. Pérez-Lorenzo, J. Dinasquet, N. Haëntjens, C. Dimier, V. Taillandier, Deep maxima of phytoplankton biomass, primary production and bacterial production in the Mediterranean Sea during late spring. Biogeosci. Discuss. 2020, 1–28 (2020).

[74] J. B. Edson, V. Jampana, R. A. Weller, S. P. Bigorre, A. J. Plueddemann, C. W. Fairall, S. D. Miller, L. Mahrt, D. Vickers, H. Hersbach, On the exchange of momentum over the open ocean. J. Phys. Oceanogr. 43, 1589–1610 (2013).

[75] D. A. Rutan, S. Kato, D. R. Doelling, F. G. Rose, L. T. Nguyen, T. E. Caldwell, N. G. Loeb, CERES synoptic product: Methodology and validation of surface radiant flux. J. Atmos. Oceanic Tech. 32, 1121–1143 (2015).

[76] A. Mahadevan, A. Pascual, D. L. Rudnick, S. Ruiz, J. Tintoré, E. D’Asaro, Coherent pathways for vertical transport from the surface ocean to interior. Bull. Am. Meteorol. Soc. 101, E1996–E2004 (2020).

