# Supplementary material for: Observations of a splitting ocean cyclone resulting in subduction of surface waters
Source: Sci Adv. 2025 Jul 23;11(30):eadu3221. doi: 10.1126/sciadv.adu3221 (PMC12285707; doi:10.1126/sciadv.adu3221)
Supplement: Supplementary file 1 — CALYPSO Collaboration authors and affiliations Supplementary Materials and Methods Supplementary Text Figs. S1 to S3 References [file sciadv.adu3221_sm.pdf]

Science Advances
AAAS

Supplementary Materials for

Observations of a splitting ocean cyclone resulting in subduction of surface waters

Leo Middleton *et al.*

Corresponding author: Leo Middleton, leo.middleton@whoi.edu; Amala Mahadevan, amahadevan@whoi.edu; Eric A. D'Asaro, dasaro@apl.washington.edu

Sci. Adv. **11**, eadu3221 (2025)
DOI: 10.1126/sciadv.adu3221

This PDF file includes:

CALYPSO Collaboration authors and affiliations
Supplementary Materials and Methods
Supplementary Text
Figs. S1 to S3
References

CALYPSO Collaboration authors and affiliations

Benjamin A. Hodges, Woods Hole Oceanographic Institution

Daniel L. Rudnick, Scripps Institution of Oceanography Craig A. Carlson, University of California, Santa Barbara Olga Matantseva, Max Planck Institute for Evolutionary Biology A. D. Kirwan Jr., University of Delaware

Irina I. Rypina, Woods Hole Oceanographic Institution

Mara A. Freilich, MIT-WHOI Joint Program; Brown University

H.M. Aravind, Northeastern University

Matthias Lankhorst, Scripps Institution of Oceanography Annalisa Griffa, CNR-ISMAR

Helga S. Huntley, Rowan University

Giuseppe Suaria, CNR-ISMAR

Giovanni Testa, CNR-ISMAR

Francesco M. Falcieri, CNR-ISMAR

Lorenzo Pasculli, CNR-ISMAR; Università Ca' Foscari, Nikolaos D. Zarokanellos, SOCIB

Maximo Garcia-Jove, SOCIB

Baptiste Mourre, SOCIB; IMEDEA (CSIC-UIB)

Mathieu Dever, Woods Hole Oceanographic Institute

Materials and Methods

Carbon estimates

During the 2022 CALYPSO cruise we took 51 bottle samples of Particulate Organic Carbon (POC), in order to evaluate chlorophyll fluorescence as a proxy for carbon concentration. We measured chlorophyll fluorescence using an ECO Puck mounted on the CTD Rosette, and compared directly the measured chlorophyll fluorescence at the sample depth to the bottle-measured POC, shown in Figure S2. The chlorophyll fluorescence was calibrated for consistency between the CTD Rosette and the underway EcoCTD profiler (*29*). Chlorophyll fluorescence is an imperfect proxy for POC, and the chlorophyll-to-carbon ratio is known to change substantially with depth due to photoacclimation and between regions due to changes in community composition. To account for these local changes in POC, we have fit a linear relationship between chlorophyll a concentration and our water sample measurements of POC taken during the cruise, that accounts for some of the variations in the chlorophyll-to-carbon ratio with depth, and accounts for the regional variations in community composition. Note that this is an attempt to use chlorophyll as a proxy for the total small-POC, not specifically living POC, which gives a larger conversion factor than when isolating living organic matter (*73*).

There is a daily cycle of photosynthesis that we have not extracted from our observations. To show the magnitude of this variation, we have plotted the average chlorophyll profiles over the daytime and nightime over the study period associated with the splitting eddy (22nd February -1st March). There is substantial variation within the photic zone, although the magnitude is less than the observed change in the chlorophyll profile before and after the splitting event, as discussed in Figure 6 of the manuscript. There is a sub-surface peak in the averaged chlorophyll profile. However, much of the sub-surface peak is due to the effects of Non-Photochemical Quenching (NPQ) reducing the measured chlorophyll fluorescence in the daytime (Fig. S2). We have estimated the depth of the photic zone using a Photosynthetically Available Radiation (PAR) sensor equipped to one of the WireWalkers (*31*) that we deployed, plotted in Figure S2. The photic zone is relatively shallow, around 40-60 m, so the effects of NPQ will not influence the measured chlorophyll at the depths where we have observed subduction, although it may play a minor role in the surface changes observed before and after splitting.

Supplementary Text

Fluxes

Although we have focused on fluxes of POC in the manuscript, we can also evaluate the fluxes of heat. In Figure S1 we have plotted the spatially averaged vertical flux as it varies in time, as well as the horizontal averaged before and after profiles for Temperature. A substantial heat flux is associated with the splitting eddy, but also heat fluxes are clearly important in the temperature front to the south of the splitting eddy, which is not resolved by our map.

Meteorological Conditions

During the study period, we measured a period of high winds during the eddy splitting process (Fig. S3c). This caused a net heat flux into the ocean, driven by latent heat fluxes, comparable to the positive heat fluxes experienced in the day time, primarily due to shortwave radiation (Fig. S3b). The heat fluxes, and wind stress associated with the wind event caused some deepening of the mixed layer depth, as defined with a threshold density value of 0.03 kg m^{-3} different from the surface. However, the mixed layer depth maintained large lateral variability during the high-winds captured in Fig. S3d. We found that without this wind event, an idealized model could still reproduce the observed splitting. However, the deepening of the mixed layer is associated with an Ekman buoyancy flux that will add to the effect of the eddy splitting on vertical fluxes. Future work will discuss the role of Ekman pumping on heat fluxes in the mixed layer during the observed splitting.

The N/O *Pourquoi Pas?* (PQP) had a BATOS weather station maintained by Meteo France, and the R/V *Pelagia* had a weather station maintained by the Koninklijk Nedelands Meteorologisch Instituut. Using the wind speed, air temperature, relative humidity, air pressure, water temperature and downward shortwave radiation measured on the PQP, we calculated the sensible and latent heat fluxes using the COARE algorithm (*74*). Shortwave radiation is measured directly by the BATOS weather station, and longwave radiation is interpolated onto the ship track from CERES synoptic product (*75*). All flux data is binned in one hour increments.

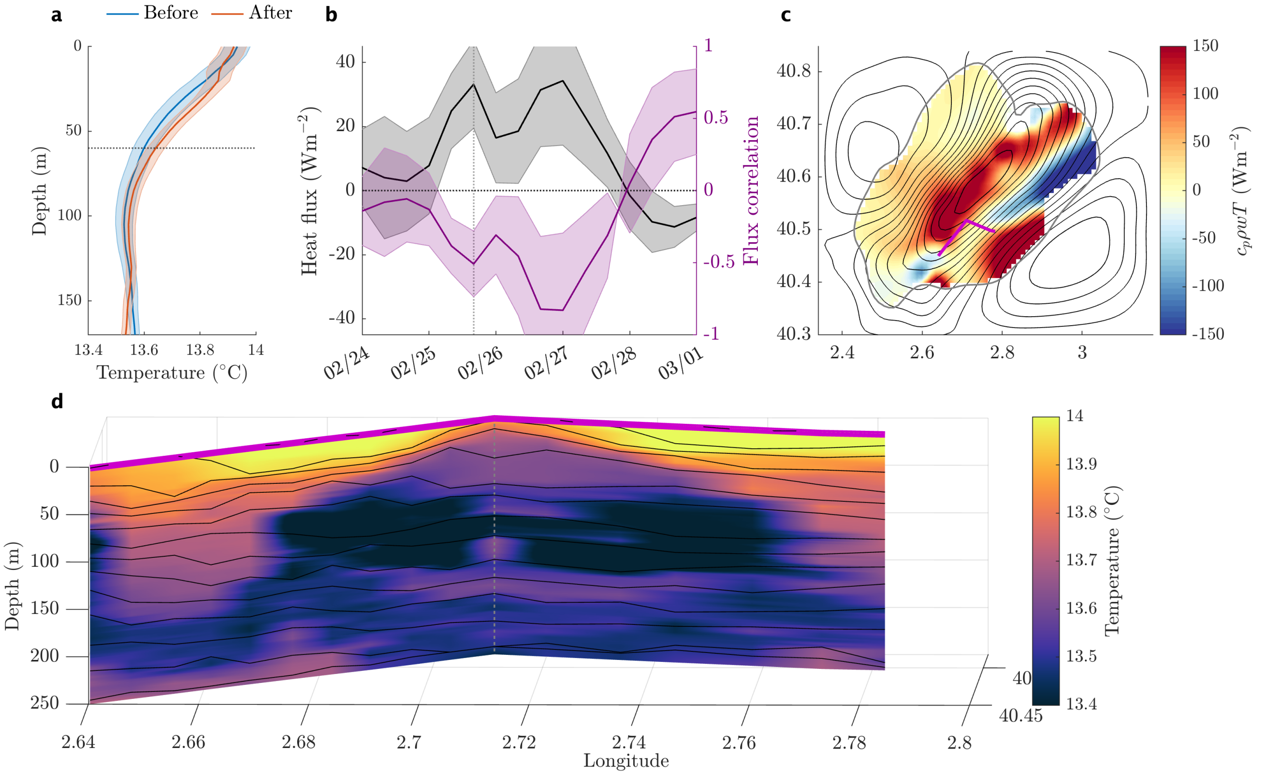

Figure S1: **The contribution of eddy splitting to vertical fluxes of heat.** (a) Horizontally averaged temperature profile, before and after the eddy splitting from the variational map. (b) Horizontally averaged vertical heat flux $\rho c_p \langle w'T' \rangle$ as it varies throughout the eddy splitting. Also included is horizontally averaged flux correlation $\langle w'T' \rangle / \langle |w| \rangle \langle |T| \rangle$. (c) The spatial variation in the vertical heat flux at 60 m depth on the 26th February. (d) Profile of temperature after the splitting of the eddy, with contours of density.

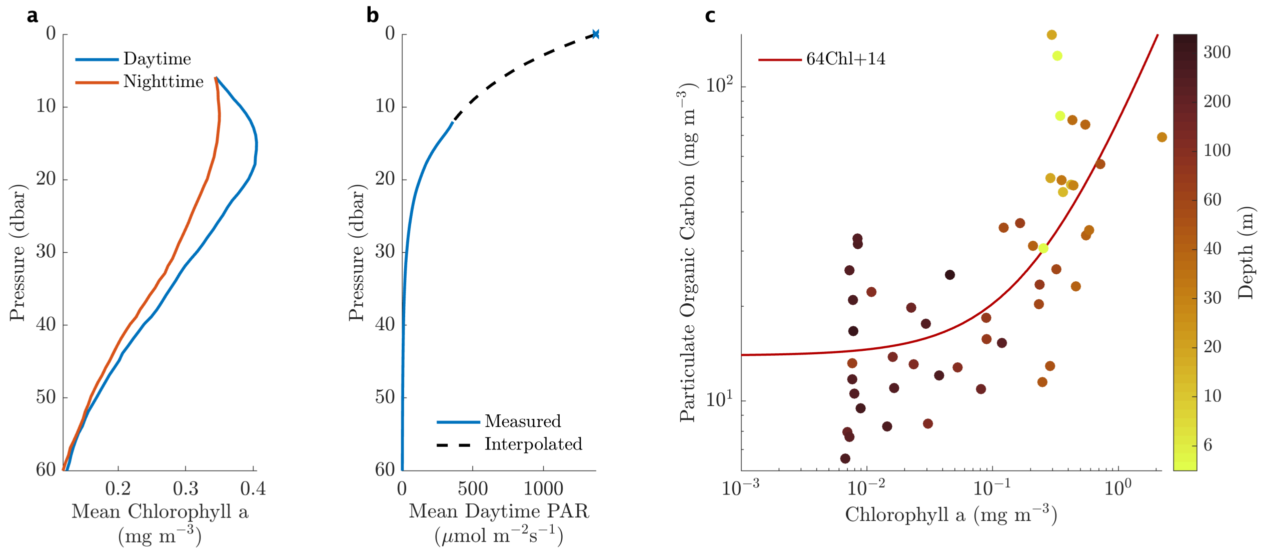

Figure S2: **The distribution of chlorophyll concentrations as compared with Particulate Organic Carbon (POC) and Photosynthetically Available Radiation (PAR).** (a) the averaged Chlorophyll profile across all the EcoCTD sampling during the eddy splitting, segmented by daytime and nighttime. (b) the averaged midday PAR from the WireWalker, with an interpolation between the surface buoy PAR sensor and the WireWalker profiles below. (c) the POC samples taken from the CTD Rosette, compared with a calibrated chlorophyll concentration from the CTD fluorometer, colored by the sample depth. We show the least-squares linear fit, appearing curved as the axes are logarithmic.

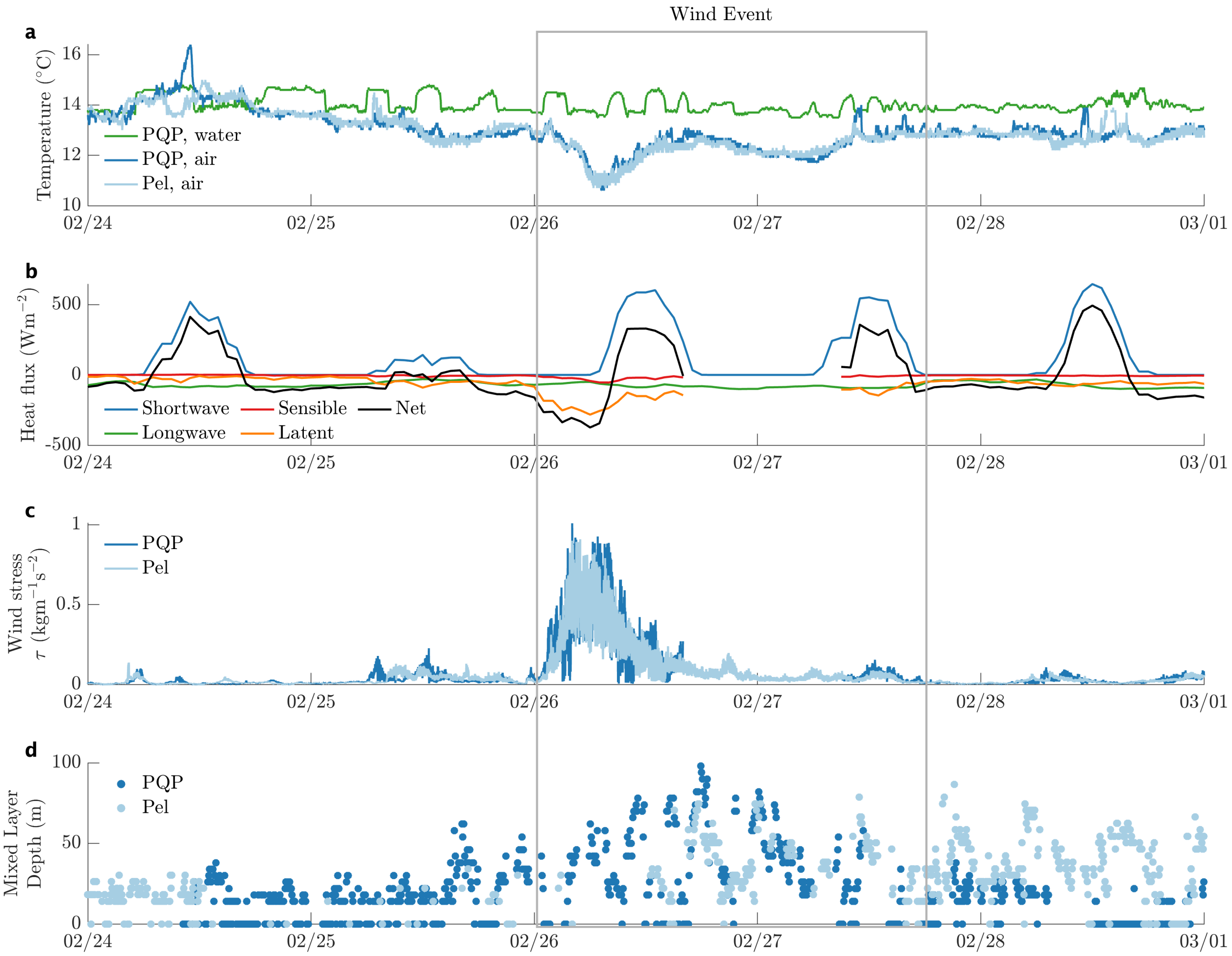

Figure S3: **Meteorological conditions during the study period.** (a) water temperature and air temperature as measured by the *N/O Pourquoi Pas?* and the *R/V Pelagia*. (b) the net ocean/atmosphere heat flux, broken into shortwave and longwave radiative parts, sensible and latent heat fluxes. (c) wind stress from the Pourquoi Pas? and Pelagia. (d) Mixed Layer Depth inferred from the Pourquoi Pas? and Pelagia.

REFERENCES AND NOTES

1. W. Munk, L. Armi, K. Fischer, F. Zachariasen, Spirals on the sea. *Proc. R. Soc. Lond. Ser. A: Math. Phys. Eng. Sci.* **456**, 1217–1280 (2000).

2. P. Scully-Power, *Navy Oceanographer Shuttle Observations, STS 41-G Mission Report* (Naval Underwater Systems Center, NUSC Technical Document 7611, 1986).

3. D. L. Rudnick, On the skewness of vorticity in the upper ocean. *Geophys. Res. Lett.* **28**, 2045–2048 (2001).

4. A. Y. Shcherbina, E. A. D'Asaro, C. M. Lee, J. M. Klymak, M. J. Molemaker, J. C. McWilliams, Statistics of vertical vorticity, divergence, and strain in a developed submesoscale turbulence field. *Geophys. Res. Lett.* **40**, 4706–4711 (2013).

5. H. Cao, M. Freilich, X. Song, Z. Jing, B. Fox-Kemper, B. Qiu, R. D. Hetland, F. Chai, S. Ruiz, D. Chen, Isopycnal submesoscale stirring crucially sustaining subsurface chlorophyll maximum in ocean cyclonic eddies. *Geophys. Res. Lett.* **51**, e2023GL105793 (2024).

6. P. Klein, G. Lapeyre, The oceanic vertical pump induced by mesoscale and submesoscale turbulence. *Ann. Rev. Mar. Sci.* **1**, 351–375 (2009).

7. D. J. McGillicuddy Jr., A. R. Robinson, D. A. Siegel, H. W. Jannasch, R. Johnson, T. D. Dickey, J. McNeil, A. F. Michaels, A. H. Knap, Influence of mesoscale eddies on new production in the Sargasso Sea. *Nature* **394**, 263–266 (1998).

8. Q. Ni, X. Zhai, C. Wilson, C. Chen, D. Chen, Submesoscale eddies in the south China Sea. *Geophys. Res. Lett.* **48**, e2020GL091555 (2021).

9. M. Lévy, P. J. S. Franks, K. S. Smith, The role of submesoscale currents in structuring marine ecosystems. *Nat. Commun.* **9**, 4758 (2018).

10. A. Mahadevan, The impact of submesoscale physics on primary productivity of plankton. *Ann. Rev. Mar. Sci.* **8**, 161–184 (2016).

11. B. Fox-Kemper, R. Ferrari, R. Hallberg, Parameterization of mixed layer eddies. Part I: Theory and diagnosis. *J. Phys. Oceanogr.* **38**, 1145–1165 (2008).

12. M. M. Omand, E. A. D'Asaro, C. M. Lee, M. J. Perry, N. Briggs, I. Cetinić, A. Mahadevan, Eddy-driven subduction exports particulate organic carbon from the spring bloom. *Science* **348**, 222–225 (2015).

13. P. W. Boyd, H. Claustre, M. Levy, D. A. Siegel, T. Weber, Multi-faceted particle pumps drive carbon sequestration in the ocean. *Nature* **568**, 327–335 (2019).

14. M. Dever, D. Nicholson, M. Omand, A. Mahadevan, Size-differentiated export flux in different dynamical regimes in the ocean. *Global Biogeochem. Cycles* **35**, e2020GB006764 (2021).

15. L. Resplandy, M. Lévy, D. J. McGillicuddy Jr., Effects of eddy-driven subduction on ocean biological carbon pump. *Global Biogeochem. Cycles* **33**, 1071–1084 (2019).

16. R. Asselot, L. I. Carracedo, V. Thierry, H. Mercier, R. Bajon, F. F. Pérez, Anthropogenic carbon pathways towards the North Atlantic interior revealed by Argo-O_2, neural networks and back-calculations. *Nat. Commun.* **15**, 1630 (2024).

17. W. Cui, W. Wang, J. Zhang, J. Yang, Multicore structures and the splitting and merging of eddies in global oceans from satellite altimeter data. *Ocean Sci.* **15**, 413–430 (2019).

18. Q.-Y. Li, L. Sun, S.-F. Lin, GEM: A dynamic tracking model for mesoscale eddies in the ocean. *Ocean Sci.* **12**, 1249–1267 (2016).

19. M. W. Schouten, W. P. de Ruijter, P. J. Van Leeuwen, J. R. Lutjeharms, Translation, decay and splitting of Agulhas rings in the southeastern Atlantic Ocean. *J. Geophys. Res. Oceans* **105**, 21913–21925 (2000).

20. D. Nof, The role of angular momentum in the splitting of isolated eddies. *Tellus A Dyn. Meteorol. Oceanogr.* **42**, 469–481 (2022).

21. H. L. Simmons, D. Nof, Islands as eddy splitters. *J. Mar. Res.* **58**, 919–956 (2000).

22. F. Fang, R. Morrow, Evolution, movement and decay of warm-core Leeuwin Current eddies. *Deep Sea Res. II Top. Stud. Oceanogr.* **50**, 2245–2261 (2003).

23. S. Wang, Z. Liu, C. Pang, Geographical distribution and anisotropy of the inverse kinetic energy cascade, and its role in the eddy equilibrium processes. *J. Geophys. Res. Oceans* **120**, 4891–4906 (2015).

24. J. M. Steinberg, S. T. Cole, K. Drushka, R. P. Abernathey, Seasonality of the mesoscale inverse cascade as inferred from global scale-dependent Eddy energy observations. *J. Phys. Oceanogr.* **52**, 1677–1691 (2022).

25. H. Hewitt, B. Fox-Kemper, B. Pearson, M. Roberts, D. Klocke, The small scales of the ocean may hold the key to surprises. *Nat. Clim. Chang.* **12**, 496–499 (2022).

26. Z. Su, J. Wang, P. Klein, A. F. Thompson, D. Menemenlis, Ocean submesoscales as a key component of the global heat budget. *Nat. Commun.* **9**, 775 (2018).

27. M. Juza, L. Renault, S. Ruiz, J. Tintoré, Origin and pathways of winter intermediate water in the northwestern mediterranean sea using observations and numerical simulation. *J. Geophys. Res. Oceans* **118**, 6621–6633 (2013).

28. D. L. Rudnick, J. Klinke, The underway conductivity–temperature–depth instrument. *J. Atmos. Oceanic Tech.* **24**, 1910–1923 (2007).

29. M. Dever, M. Freilich, J. T. Farrar, B. Hodges, T. Lanagan, A. J. Baron, A. Mahadevan, EcoCTD for profiling oceanic physical–biological properties from an underway ship. *J. Atmos. Oceanic Tech.* **37**, 825–840 (2020).

30. R. Davis, J. Sherman, J. Dufour, Profiling ALACEsdd other advances in autonomous subsurface floats. *J. Atmos. Oceanic Tech.* **18**, 982–993 (2001).

31. R. Pinkel, M. Goldin, J. Smith, O. Sun, A. Aja, M. Bui, T. Hughen, The Wirewalker: A vertically profiling instrument carrier powered by ocean waves. *J. Atmos. Oceanic Tech.* **28**, 426–435 (2011).

32. P.-M. Poulain, L. Centurioni, T. Özgökmen, Comparing the currents measured by CARTHE, CODE and SVP drifters as a function of wind and wave conditions in the southwestern Mediterranean Sea. *Sensors* **22**, 353 (2022).

33. R. Lumpkin, M. Pazos, Measuring surface currents with Surface Velocity Program drifters: The instrument, its data, and some recent results. *LAPCOD* **39**, 67 (2007).

34. R. E. Davis, Drifter observations of coastal surface currents during CODE: The method and descriptive view. *J. Geophys. Res. Oceans* **90**, 4741–4755 (1985).

35. G. Novelli, C. M. Guigand, C. Cousin, E. H. Ryan, N. J. Laxague, H. Dai, B. K. Haus, T. M. Özgökmen, A biodegradable surface drifter for ocean sampling on a massive scale. *J. Atmos. Oceanic Tech.* **34**, 2509–2532 (2017).

36. A. Barth, J.-M. Beckers, C. Troupin, A. Alvera-Azcárate, L. Vandenbulcke, divand-1.0: *n*-dimensional variational data analysis for ocean observations. *Geosci. Model Dev.* **7**, 225–241 (2014).

37. E. Cutolo, A. Pascual, S. Ruiz, T. Shaun Johnston, M. Freilich, A. Mahadevan, A. Shcherbina, P.-M. Poulain, T. Ozgokmen, L. R. Centurioni, D. L. Rudnick, E. D'Asaro, Diagnosing frontal dynamics from observations using a variational approach. *J. Geophys. Res. Oceans* **127**, e2021JC018336 (2022).

38. M. Arai, T. Yamagata, Asymmetric evolution of eddies in rotating shallow water. *Chaos* **4**, 163–175 (1994).

39. S. Kida, Motion of an elliptic vortex in a uniform shear flow. *J. Physical Soc. Japan* **50**, 3517–3520 (1981).

40. J. Dingwall, T. Chor, J. R. Taylor, Large eddy simulations of the accumulation of buoyant material in oceanic wind-driven and convective turbulence. *J. Fluid Mech.* **954**, A27 (2023).

41. J. R. Holton, G. J. Hakim, *An introduction to dynamic meteorology* (Academic press, 2012).

42. C. J. Shakespeare, Curved density fronts: Cyclogeostrophic adjustment and frontogenesis. *J. Phys. Oceanogr.* **46**, 3193–3207 (2016).

43. D. A. Schecter, M. T. Montgomery, On the symmetrization rate of an intense geophysical vortex. *Dyn. Atmos. Oceans* **37**, 55–88 (2003).

44. C. Gao, P. Zhu, Vortex Rossby wave propagation in baroclinic tropical cyclone-like vortices. *Geophys. Res. Lett.* **43**, 12–578 (2016).

45. M. Melander, J. McWilliams, N. Zabusky, Axisymmetrization and vorticity-gradient intensification of an isolated two-dimensional vortex through filamentation. *J. Fluid Mech.* **178**, 137–159 (1987).

46. A. Ioannou, A. Stegner, A. Tuel, B. LeVu, F. Dumas, S. Speich, Cyclostrophic corrections of AVISO/DUACS surface velocities and its application to mesoscale eddies in the Mediterranean Sea. *J. Geophys. Res. Oceans* **124**, 8913–8932 (2019).

47. J. R. Taylor, A. F. Thompson, Submesoscale dynamics in the upper ocean. *Annu. Rev. Fluid Mech.* **55**, 103–127 (2023).

48. H. Loisel, A. Morel, Light scattering and chlorophyll concentration in case 1 waters: A reexamination. *Limnol. Oceanogr.* **43**, 847–858 (1998).

49. D. L. Rudnick, N. D. Zarokanellos, J. Tintoré, A four-dimensional survey of the Almeria–Oran Front by underwater gliders: tracers and circulation. *J. Phys. Oceanogr.* **52**, 225–242 (2022).

50. D. R. Tarry, S. Ruiz, T. S. Johnston, P.-M. Poulain, T. Özgökmen, L. R. Centurioni, M. Berta, G. Esposito, J. T. Farrar, A. Mahadevan, Drifter observations reveal intense vertical velocity in a surface ocean front. *Geophys. Res. Lett.* **49**, e2022GL098969 (2022).

51. M. A. Spall, Frontogenesis, subduction, and cross-front exchange at upper ocean fronts. *J. Geophys. Res. Oceans* **100**, 2543–2557 (1995).

52. M. Freilich, A. Mahadevan, Coherent pathways for subduction from the surface mixed layer at ocean fronts. *J. Geophys. Res. Oceans* **126**, e2020JC017042 (2021).

53. R. Ferrari, C. Wunsch, Ocean circulation kinetic energy: Reservoirs, sources, and sinks. *Annu. Rev. Fluid Mech.* **41**, 253–282 (2009).

54. D. Balwada, J.-H. Xie, R. Marino, F. Feraco, Direct observational evidence of an oceanic dual kinetic energy cascade and its seasonality. *Sci. Adv.* **8**, eabq2566 (2022).

55. K. Srinivasan, R. Barkan, J. C. McWilliams, A forward energy flux at submesoscales driven by frontogenesis. *J. Phys. Oceanogr.* **53**, 287–305 (2023).

56. M. Freilich, L. Lenain, S. T. Gille, Characterizing the role of non-linear interactions in the transition to submesoscale dynamics at a dense filament. *Geophys. Res. Lett.* **50**, e2023GL103745 (2023).

57. J. C. McWilliams, Submesoscale, coherent vortices in the ocean. *Rev. Geophys.* **23**, 165–182 (1985).

58. D. McCoy, D. Bianchi, A. L. Stewart, Global observations of submesoscale coherent vortices in the ocean. *Prog. Oceanogr.* **189**, 102452 (2020).

59. E. A. D'Asaro, Generation of submesoscale vortices: A new mechanism. *J. Geophys. Res. Oceans* **93**, 6685–6693 (1988).

60. J. A. MacKinnon, H. L. Simmons, J. Hargrove, J. Thomson, T. Peacock, M. H. Alford, B. I. Barton, S. Boury, S. D. Brenner, N. Couto, S. L. Danielson, E. C. Fine, H. C. Graber, J. Guthrie, J. E. Hopkins, S. R. Jayne, C. Jeon, T. Klenz, C. M. Lee, Y. D. Lenn, A. J. Lucas, B. Lund, C. Mahaffey, L. Norman, L. Rainville, M. M. Smith, L. N. Thomas, S. Torres-Valdés, K. R. Wood, A warm jet in a cold ocean. *Nat. Commun.* **12**, 2418 (2021).

61. C. E. Buckingham, J. Gula, X. Carton, The role of curvature in modifying frontal instabilities. Part II: Application of the criterion to curved density fronts at low Richardson numbers. *J. Phys. Oceanogr.* **51**, 317–341 (2021).

62. E. Pallàs-Sanz, T. Johnston, D. Rudnick, Frontal dynamics in a California current system shallow front: 2. Mesoscale vertical velocity. *J. Geophys. Res. Oceans* **115**, C12068 (2010).

63. A. Mahadevan, E. A. D'Asaro, Cruise report: Calypso, R/V Pourquoi Pas? (OSF, 2022).

64. T. M. S. Johnston, N. Calafat, B. Casas, E. Cutolo, R. Daniels, F. Falcieri, T. Litchendorf, I. Lizarán, C. McNeil, A. Pascual, Cruise report: Calypso, R/V Pelagia, Cruise No. 64pe497 (OSF, 2022); doi:10.17605/OSF.IO/EN7T4.

65. C. Troupin, A. Barth, D. Sirjacobs, M. Ouberdous, J.-M. Brankart, P. Brasseur, M. Rixen, A. Alvera-Azcárate, M. Belounis, A. Capet, Generation of analysis and consistent error fields using the Data Interpolating Variational Analysis (DIVA). *Ocean Model.* **52**, 90–101 (2012).

66. C. A. De Moura, C. S. Kubrusly, The courant–friedrichs–lewy (cfl) condition. *AMC* **10**, (2013).

67. A. Ramadhan, G. Wagner, C. Hill, J.-M. Campin, V. Churavy, T. Besard, A. Souza, A. Edelman, R. Ferrari, J. Marshall, Oceananigans. jl: Fast and friendly geophysical fluid dynamics on GPUs. *J. Open Sourc. Softw.* **5**, (2020).

68. J. Marotzke, R. Giering, K. Q. Zhang, D. Stammer, C. Hill, T. Lee, Construction of the adjoint MIT ocean general circulation model and application to Atlantic heat transport sensitivity. *J. Geophys. Res. Oceans* **104**, 29529–29547 (1999).

69. X.-D. Liu, S. Osher, T. Chan, Weighted essentially non-oscillatory schemes. *J. Comput. Phys.* **115**, 200–212 (1994).

70. W. R. Young, An exact thickness-weighted average formulation of the Boussinesq equations. *J. Phys. Oceanogr.* **42**, 692–707 (2012).

71. Visible and Infrared Imager/Radiometer Suite (VIIRS), NASA goddard space flight center, ocean ecology laboratory, ocean biology processing group. (NASA OB.DAAC, 2022).

72. B. D. Beckley, N. P. Zelensky, S. A. Holmes, F. G. Lemoine, R. D. Ray, G. T. Mitchum, S. D. Desai, S. T. Brown, Assessment of the Jason-2 extension to the TOPEX/Poseidon, Jason-1

sea-surface height time series for global mean sea level monitoring. *Mar. Geod.* **33**, 447–471 (2010).

73. E. Marañón, F. Van Wambeke, J. Uitz, E. S. Boss, M. Pérez-Lorenzo, J. Dinasquet, N. Haëntjens, C. Dimier, V. Taillandier, Deep maxima of phytoplankton biomass, primary production and bacterial production in the Mediterranean Sea during late spring. *Biogeosci. Discuss.* **2020**, 1–28 (2020).

74. J. B. Edson, V. Jampana, R. A. Weller, S. P. Bigorre, A. J. Plueddemann, C. W. Fairall, S. D. Miller, L. Mahrt, D. Vickers, H. Hersbach, On the exchange of momentum over the open ocean. *J. Phys. Oceanogr.* **43**, 1589–1610 (2013).

75. D. A. Rutan, S. Kato, D. R. Doelling, F. G. Rose, L. T. Nguyen, T. E. Caldwell, N. G. Loeb, CERES synoptic product: Methodology and validation of surface radiant flux. *J. Atmos. Oceanic Tech.* **32**, 1121–1143 (2015).

76. A. Mahadevan, A. Pascual, D. L. Rudnick, S. Ruiz, J. Tintoré, E. D'Asaro, Coherent pathways for vertical transport from the surface ocean to interior. *Bull. Am. Meteorol. Soc.* **101**, E1996–E2004 (2020).
